# Taphonomic variation in vascular remains from Mesozoic non-avian dinosaurs

**DOI:** 10.1038/s41598-025-85497-y

**Published:** 2025-02-05

**Authors:** M. H. Schweitzer, W. Zheng, E. Dickinson, J. Scannella, A. Hartstone-Rose, P. Sjövall, J. Lindgren

**Affiliations:** 1 Department of Biological Sciences, North Carolina State University, Raleigh, NC USA; 2 North Carolina Museum of Natural Sciences, Raleigh, NC USA; 3 Museum of the Rockies, Montana State University, Bozeman, MT USA; 4 Department of Geology, Lund University, Lund, Sweden; 5 Department of Anatomy, New York Institute of Technology College of Osteopathic Medicine, Old Westbury, NY USA; 6 Department of Anthropology and Archaeology, University of Calgary, Calgary, AB Canada; 7 Department of Earth Sciences, Montana State University, Bozeman, MT USA; 8 Materials and Production, RISE Research Institutes of Sweden, Borås, Sweden

**Keywords:** Ancient proteins, Dinosaur soft tissues, Immunohistochemistry, Molecular palaeontology, ToF-SIMS, Biochemistry, Evolution, Immunology, Microbiology, Molecular biology, Biogeochemistry, Materials science

## Abstract

The identity and source of flexible, semi-transparent, vascular-like components recovered from non-avian dinosaur bone are debated, because: (1) such preservation is not predicted by degradation models; (2) taphonomic mechanisms for this type of preservation are not well defined; and (3) although support for molecular endogeneity has been demonstrated in select specimens, comparable data are lacking on a broader scale. Here, we use a suite of micromorphological and molecular techniques to examine vessel-like material recovered from the skeletal remains of six non-avian dinosaurs, representing different taxa, depositional environments and geological ages, and we compare the data obtained from our analyses against vessels liberated from extant ostrich bone. The results of this in-depth, multi-faceted study present strong support for endogeneity of the fossil-derived vessels, although we also detect evidence of invasive microorganisms.

**Supplementary Information:**

The online version contains supplementary material available at 10.1038/s41598-025-85497-y.

## Introduction

Paleontologists traditionally rely on anatomical information obtained from the fossilized hard-parts of extinct animals to gain information regarding the evolution of the biosphere. However, the rock record is also capable of exceptional preservation, and occasionally, normally decay-prone organs and tissues are conserved across deep time with a high degree of morphological fidelity (reviewed in^1^). Such findings yield information on traits that are generally not available to the scientific community, and are thus instrumental for increasing our understanding of evolutionary innovations, as well as the biology of extinct organisms.

Reports of the recovery of microstructures consistent with original cells and tissues in deep-time fossils (> 1 MY) are continuously increasing (e.g.,^2–15^) and a growing body of data suggests that the preservation of such ‘soft’ (i.e., originally un-biomineralised) tissues and the molecules that once characterised them may be more common than previously thought (reviewed in^1,16^). Organ-, tissue- and cell-like structures morphologically consistent with their extant counterparts, including skin (e.g.,^10,17–26^), scales, feathers or feather-like appendages (e.g.,^12,27–34^) osteocytes, chondrocytes and other cells (e.g.,^4,35–40^) have now been reported from numerous Cenozoic and Mesozoic vertebrate fossils. Molecular data derived from these specimens could potentially revolutionize our understanding of the ancient past^41,42^.

There is broad consensus within the paleontological community regarding the preservation of recalcitrant polymers (e.g., melanic pigments) and their diagenetic transformation products in the geologic record^43–47^. In addition to pigments^3,8,9,11,48–55^, evidence for preserved blubber^10^ and proteins, including hemoglobin^41,48^, collagen (e.g.,^8,26,56–60^) and keratin^11,12,61–65^ have also been reported from deep-time fossils. However, there is far less agreement as to whether DNA, a more informative, but more degradation-prone biomacromolecule, can survive across deep time (i.e., > 1 Ma) (e.g.,^66–69^).

There is an inverse (and almost ironic) relationship between the amount of information encoded in a biomolecule and its ability to persist over geological time (e.g.,^70^). DNA carries the most biological information, yet nucleic acid chains are proposed to have the lowest preservation potential of all organic macromolecules because of their chemically fragile helical backbone^71–73^ (but see also^6,74^). Thus, studies using ancient DNA are currently limited to the last ~ 2 million years. At the other end of the spectrum, some of the most durable biomacromolecules are lipids and polymers with thermally stabile carbon skeletons, such as lignin. The organic source of these molecules is not questioned; however, although relatively well-represented in the fossil record, they are only specific to a particular organism at a very general level^69^. Nonetheless, the methodological advances in molecular biology and analytical biochemistry have provided increasing access to a novel source of geobiologically relevant information: primary organic compounds recovered from body fossils^10,75–79^.

Attempts to investigate proteinaceous residues in animal remains older than a few million years have so far been beset with obstacles, including: (1) difficulties in identification due to diagenetic alteration; (2) a lack of relevant database information for taxa that are phylogenetically distant from living animals; (3) an absence of standardised analytical protocols to exclude alternative (contaminant) sources for the detected peptides; (4) discrepancies between kinetic models of biomolecule degradation and empirical data from fossils; and (5) an incomplete understanding of stabilisation mechanisms that could explain the conservation of informative biomolecules in multimillion-year-old fossils^66,68,80–82^. These difficulties have led to a diversity of opinions regarding what is actually preserved. Additionally, questions as to how the recovered components have been modified from their original composition and structure, what information can be gained from such molecular “fossils”, and how these can be characterised are still being debated^68,83–86^. This controversy is unfortunate because soft tissues and the molecules comprising them, if properly analysed and interpreted, hold great potential for advancing our understanding of molecular evolution into deep time.

Although a growing body of data supports the preservation of original peptides in fossils many millions of years old (e.g.,^14,15,87–89^), skepticism persists because some researchers consider the constituent amino acid chains to be either too fragile to withstand the test of time (e.g.,^90–92^), or inversely, converted into highly condensed and mostly defunctionalised geomolecules (e.g.,^55,93^). Therefore, to test whether purportedly degradation-prone tissues and biomolecules (proteins) under certain conditions can survive across deep time, we compared the morphological and chemical composition of hollow, vascular-like microstructures (hereafter ‘vessels’ for space and simplicity) isolated from six non-avian dinosaurs and an extant ostrich (see Supplementary Information and Supplementary Table S1). The fossil material included phylogenetically distinct taxa (i.e., ornithischian and saurischian dinosaurs), and differed in geographic location, depositional setting and geological age. We employed multiple analytical methods to determine the histological integrity and molecular composition of the vessels, reasoning that if endogenous, they should display at least some similarities with extant, bone-derived vessels. We used data obtained from these Mesozoic vessels to test the following hypotheses: (1) the vessels are endogenous to the dinosaurs from which they derive; (2) depositional environments are predictors of vascular preservation; and (3) vascular integrity in fossils is not dependent upon geological age or taxon.

Our comprehensive experimental approach revealed a complex mixture of endogenous dinosaur-derived organics, mineral coatings/replacement structures and invasive microorganisms, with implications not only for the taphonomy of vascular tissues but also for inferences on potential phylogenetic, metabolic and physiological signals retained in vertebrate hard tissues (e.g.,^55,93,94^).

### Composition and structure of extant blood vessels

Blood vessels in living organisms are composed of three main layers, including (from the lumen to exterior): an endothelial lining (tunica intima), a smooth muscle layer (tunica media) and external connective tissues (tunica adventitia or externa; Supplementary information, Fig. S1)^95^. Endothelial cell cytoplasmic extensions are normally very thin, but joined by tight junctions to form the tunica intima^96^. Endothelial cell nuclei protrude into the vascular lumen^97^, and contain a full complement of DNA^98^. Endothelial cells also produce a basement membrane that forms a relatively impermeable layer in most vessels^95^; this membrane is made of structural proteins, including elastin and laminin^99–103^. Laminin contributes to the impermeability of the vessel wall^104^, while elastin provides resilience, thereby allowing vessels to respond to blood pressure changes (e.g.,^105^). Notably, this latter, long-lasting protein^106,107^ contains the amino acid desmosine that is unique to elastin protein^108,109^. Therefore, the localisation of antibodies to laminin, elastin and/or desmosine to the wall of these ancient vessels provides strong evidence for endogeneity of these vascular remains.

External to the endothelium and its basement membrane is a layer of smooth muscle cells. This may consist of a single layer of cells in the smallest vessels to multiple layers in larger vessels, forming the tunica media; thus, standard contractile proteins (myosin, tropomyosin) are localised to this layer^95,110,111^. Elastin is also secreted by smooth muscle cells of the tunica media^98,112^.

The outermost layer of vessels, the tunica adventitia, is comprised of connective tissues. This layer is rich in fibrillar collagens, but elastin, nerves, small capillaries, and glycosaminoglycans are also distributed here^95,98,107,109,113^.

### Rationale for experimental approach to characterise ancient organic remains

Because extant vessels are heterogeneous and are comprised of different proteins, this knowledge provided us with analytical targets, depending on the layer being analysed (Supplementary Information Fig. S1). We predicted that although all layers may not be represented, or may not be readily distinguished in 80–66 million-year-old dinosaur vessels, at least some of these components should be detectable in fossilised vascular remains, if endogenous. We further predicted that it would be likely for these vessels to also exhibit a microbial or fungal component, either ancient or modern, after millions of years in geochemically active environments. Therefore, we chose analytical methods that could discriminate between these sources.

## Materials and methods

### Blood vessel collection

Cortical bone fragments, collected from intact bone during excavation (MOR 1125, MOR 2598, MOR 10857) or from smaller fragments isolated during excavation and/or preparation (MOR 555/USNM 555000, MOR 1126, MOR 1128) from four specimens of *Tyrannosaurus rex* (MOR 555/USNM 555000, MOR 1125, MOR 1126, MOR 1128), *Brachylophosaurus canadensis* (MOR 2598), and an indeterminate ceratopsid (MOR 10857) (Table S1) were demineralised in ethylenediaminetetra-acetic acid (EDTA) (500 mM, pH 8.0) for at least 2 weeks with daily buffer changes. Liberated vessels were collected and washed with e-pure water to remove residual EDTA for LM imaging and further treatment.

Small fragments of ostrich long bone (excised 9/26/2004, incubated with 10% Zout (Dial Corp.) to defat, then stored under desiccation with an antifungal powder for approximately 17 years) were demineralised with 0.5 M EDTA pH 8.0 until most of the mineral phase was removed and remaining tissues were pliable. Demineralised bone was then cut into 0.5–1 mm slices with a sterile razor blade, and washed with e-pure water ~ 10 times to completely remove EDTA. Following this treatment, bone slices were digested with 1 mg/mL collagenase A (Roche10103578001) in Dulbecco’s phosphate-buffered saline (D-PBS) pH 7.2 (with 0.1 g/L calcium chloride and 0.1 g/L magnesium chloride added) at 37 °C overnight or until the collagen matrix was completely removed. The remaining vessels were collected under magnification and washed with e-pure water to remove residual collagenase, then incubated for 1 h at room temperature in neutral buffered 10% formalin for embedding.

### Transmitted light microscopy (LM)

Vessels liberated and prepared as above were examined with a Zeiss Stemi-2000-C or a Zeiss Axioskop 2 plus biological microscope, and images were captured using an AxioCam MRc 5 (Zeiss) in the Axiovision software package (version 4.7.0.0).

### Scanning electron microscopy (SEM)

Ancient vessels and 10% formalin fixed ostrich vessels were dehydrated with a graded ethanol series. Then, the specimens were transported to the Chapel Hill Analytical and Nanofabrication Laboratory (CHANL) core facility where they were subjected to critical point drying (Tousimis Semidri PVT-3) and sputter coated (Cressington 108 Auto) with ~ 70 angstroms of palladium gold. Prepared samples were then analysed using a CHANL Hitachi S-4700 Cold Cathode Field Emission Gun Scanning Electron Microscope (FEG-SEM) at an accelerating voltage of 5kEV.

In addition to vessels recovered as above, we also used vessels from *Tyrannosaurus rex* (MOR 1125) that were collected and lyophilised in 2006 and stored at − 90 °C until use. They were brought to room temperature in PBS, mounted on stubs with double-sided carbon tape and also analysed with SEM as above.

### Transmission electron microscopy (TEM)

Isolated vessels from each dinosaur specimen were incubated in a solution of pyridoxal isonicotinoyl hydrazone (PIH) (5 mM PIH in 50 mM NaOH)^114^ overnight at room temperature to chelate excess iron from the recovered tissues, then washed with e-pure water. PIH-treated ancient vessels and 10% formalin fixed ostrich vessels were first embedded in 3% agar to stabilize the tissues, followed by embedding in LR White resin after partial dehydration in 70% ethanol and infiltration with pure LR White water permeable embedding medium as previously described^80,115^. 90-nm-thick sections were cut on a Leica EM UC6 Ultramicrotome, mounted on carbon-coated nickel grids (EMS Cat CFT200-NI), and stained with 5% methanolic uranyl acetate for 5 min and Reynold’s lead citrate for 8 min, and then observed using the Talos F200X G2 electron microscope in the Analytical Instrumentation Facility (AIF) of North Carolina State University.

### Nano computed tomography (nano-CT)

Because ostrich vessels are not mineralised in life, they are difficult to visualise without staining. Therefore, we stained these extant vessels with an aqueous 2.5% I_2_KI solution (I_2_KI: one part iodine to two parts potassium iodide in an aqueous solution) for 2 × 72 h. To produce a master solution of 5% I2KI, we first dissolved 10% w/v potassium iodide into e-pure water until clear, then added 5% w/v elemental iodine into the same solution. We then diluted the master solution to 2.5% I2KI with pure water.

Ancient vessels collected as above and stained ostrich vessels were embedded and stabilised with 3% agar in 1.5 ml centrifuge tubes, scanned at a voltage of 80 kV and a power of 7w with Zeiss Xradia 510 Versa 3D X-ray Tomography System in the Analytical Imaging Facility (AIF) of North Carolina State University.

### Immunohistochemistry

#### Immunofluorescence

200-nm-thick sections of LR white embedded vessels, prepared as described above, were taken on a Leica EM UC6 Ultramicrotome and dried overnight at 45°C to each well of a Teflon coated slide (Electron Microscopy Sciences). Antigen retrieval and quenching of autofluorescence were accomplished by etching with Proteinase K (PCR grade, Roche, 25 ug/ml) in 1× phosphate buffered saline (PBS) at 37 °C for 15 min, incubated in 500 mM EDTA pH 8.0 for 30 min, followed by two incubations in 1 mg/ml sodium borohydride (NaBH_4_) for 10 min each. Incubations were interrupted by two 5 min washes in PBS. Spurious binding was inhibited by 4% normal goat serum (NGS) applied to sections for 2 h at room temperature. Sections were incubated overnight at 4 °C in either primary antibody (Table S2A) diluted to final concentration in primary dilution buffer, consisting of 1% bovine serum albumin (BSA) (Fisher, BP1660-100), 0.1% cold fish skin gelatin (Sigma G7765), 0.05% sodium azide (Sigma S-8032), and 0.01 M PBS pH 7.2), or in primary dilution buffer without added antibodies to control for non-specific binding of secondary antibody. Anti-elastin and anti-desmosine antibodies were also diluted to 1:75 and incubated with 16 mg of elastin or 10 mg desmosine (Table S2B) to inhibit binding by blocking the antibody binding site. These blocked antibodies were then applied to sections as above, to test the specificity of those antibodies. All sections, including controls, were incubated for 2 h in secondary antibody (biotinylated goat anti-rabbit IgG(H + L) (Vector BA-1000) diluted 1:500 for rabbit primary antibodies, biotinylated goat anti-mouse IgG (H + L) (Vector BA-9200), diluted 1:500 for monoclonal mouse anti-peptidoglycan. Sections were then incubated with Fluorescein Avidin D (FITC, Vector Laboratories A-2001) for 1 h at RT. All incubations were interrupted by sequential washes (2 × 10 min each) in PBS w/Tween 20, followed by two 10 min rinses in PBS. Finally, sections were mounted with Vectashield H-1000 mounting media, and coverslips were applied. Sections were examined with Zeiss Axioskop 2 plus biological microscope and captured using an AxioCam MRc 5 (Zeiss) with ×10 ocular magnification on the Axioskop 2 plus in the Axiovision software package (version 4.7.0.0).

#### Immunogold labeling (IG)

Ancient vessels and extant ostrich vessels were also subjected to immunogold (IG) labeling to demonstrate antibody-antigen complexes on tissues at higher resolution. 90-nm-thick sections of LR White embedded vessels (described above) were collected on carbon-coated nickel grids (EMS Cat CFT200-NI), incubated on droplets of PBS-Tween 20 for 10 min. 5% Normal Donkey serum (NDS) was applied to occupy non-specific binding sites and prevent spurious binding, and allowed to incubate for 1 h at room temperature. Sections on grids were then incubated with primary antibody, diluted 1:10 in primary dilution buffer as described, for 3 h at room temperature. Sections were rinsed with TBS-Tween for 10 × 2 min. All grids were then incubated with secondary antibodies (12 nm Colloidal Gold AffiniPure Donkey Anti-Rabbit IgG (H + L) 1:20 (Jackson Immuno Research Inc., Cat 715-205-152) in secondary dilution buffer for 1 h. Grids were then rinsed with PBS-Tween20 for 10 × 2 min, followed by E-pure water rinses 3 × 30 s and dried with filter paper. Sections were stained with 5% methanolic uranyl acetate for 5 min and Reynold’s lead citrate for 8 min, then observed using the Talos F200X G2 electron microscope in AIF of North Carolina State University.

#### Lactophenol cotton blue (LPCB) staining

Ancient vessels and several samples of pond fungi/biofilm were stained with LPCB to detect fungal influence and to differentiate vascular structures from invading fungal hyphae. A drop of Lactophenol Cotton Blue Solution (Sigma 61335) was placed on a glass slide. Vessels from each dinosaur, isolated and recovered as above, were transferred to the stain droplet. The samples were then covered with a coverslip. After ~ 5 min, slides were examined with Zeiss Axioskop 2 plus biological microscope and captured using an AxioCam MRc 5 (Zeiss) with ×10 ocular magnification on the Axioskop 2 plus in the Axiovision software package (version 4.7.0.0).

### Time-of-flight secondary ion mass spectrometry (ToF-SIMS) combined with SEM

ToF-SIMS provides spatially-resolved mass spectrometry data of solid surfaces by bombarding the sample with high-energy (primary) ions in a focused beam, and analysing the emitted (secondary) ions using a time-of-flight mass analyser. By scanning the primary ion beam across the sample surface and acquiring separate mass spectra from each pixel, the results can be presented as ion images showing the lateral distribution of secondary ions associated to specific molecular structures, or mass spectra from selected regions of interest (ROIs) within the analysis area.

Dinosaur vessels prepared by demineralisation in EDTA and suspended in deionised water (as above) were deposited on clean silicon wafer substrates using a pipette and allowed to air dry. They were then immediately mounted on the sample holder and introduced into the ToF-SIMS instrument. ToF-SIMS analyses were conducted in a TOFSIMSIV instrument (IONTOF GmbH, Münster, Germany) using 25 keV Bi_3_^+^ primary ions and low-energy electron flooding for charge compensation. Positive and negative ion data were acquired in the static SIMS regime with the instrument optimised for either high mass resolution (m/Δm = 3000–5000, lateral resolution 3–5 μm) or high lateral resolution (m/Δm = 300, lateral resolution 0.5–1 μm). Only positive ion data is presented here, as the negative ion data only provides limited information about the protein molecular structure (^116^). Mass calibration of the positive ion spectra was done using CH_3_^+^, C_2_H_3_^+^, C_3_H_3_^+^, C_4_H_5_^+^, and C_5_H_5_^+^. Reference ToF-SIMS spectra of pure protein samples were acquired at high mass resolution of collagen, elastin, hemoglobin and keratin (Sigma-Aldrich).

Assignments of peaks in the fossil spectra to the major N-containing organic ions characteristic of proteins; i.e., CH_4_N^+^, C_2_H_6_N^+^, C_4_H_8_N^+^, C_4_H_10_N^+^, C_5_H_10_N^+^, C_5_H_12_N^+^, and C_8_H_10_N^+^ (^116–119^), were based on close *m/z* agreement with the corresponding peaks in the protein reference spectra and with the theoretical ion mass values (Supplementary Information Fig. S7a). The ion images (Fig. 5a, b and Supplementary Information, Fig. S8a, b–12a, b) show the added signal intensities for Fe^+^+FeOH^+^+Fe_3_O_3_^+^ in red (representing iron oxide/phosphate), CH_4_N^+^+C_2_H_6_N^+^+C_4_H_8_N^+^+C_5_H_10_N^+^+C_5_H_12_N^+^ in green (representing proteins), and Si^+^+SiOH^+^ in blue (representing the silicon substrate).

Principal components analysis (PCA) included normalized peak intensities of the protein-associated ions in spectra from the protein-rich ROIs on the fossil sample surfaces and protein references, and was done with the Solo software (version 7.9, Eigenvector Research, Inc., USA) using Poisson scaling and mean centering as pre-preparation. Peak intensities of the protein-associated ions were normalised to the added intensities of the included peaks, prior to PCA as well as for the intensity distributions presented in Supplementary Information, Fig. S7b, c.

After ToF-SIMS analysis, the fossil samples were coated with a 15-nm-thick conducting Au/Pd film and imaged in a Zeiss Supra 40VP FEG-SEM microscope at an electron energy of 2 keV using an Everhardt-Thornley type secondary electron detector (SE2). Navigation to the areas analyzed by ToF-SIMS was accomplished using overview LM images of the sample surfaces and saved coordinates for the ToF-SIMS measurements. FEG-SEM micrographs were combined with ToF-SIMS ion images of the same sample areas using PowerPoint (Microsoft Inc., USA). The overlapping images were generated by placing semi-transparent ion images on top of the FEG-SEM micrographs, and then adjusting the size and orientation of the ion image manually to obtain a good match using obvious structural features/landmarks in the FEG-SEM and ToF-SIMS images. However, topographical variations on the sample surfaces in combination with the 45° incident angle of the primary ion beam of the ToF-SIMS imaging measurement sometimes resulted in minor mismatches in parts of the combined images.

## Results

### LM

Under LM, both modern and fossil vessels showed intact, cohesive walls surrounding a hollow lumen (Supplementary Information, Fig. S2a-u). Furthermore, most dinosaur vessels maintained interconnectivity and demonstrated tapering branches (Supplementary Information, Fig. S2a, e, l, r, u, arrows), and, in some cases, anastomoses (Supplementary Information, Fig. S2f, h, u, arrowheads), consistent with extant vasculature^120,121^. Regardless of taxon, geological age and depositional environment (Supplementary Information, Table S1), all vessels were additionally preserved in three dimensions, and exhibited both translucent and opaque regions, as well as occasional intravascular contents.

### SEM

**Fig. 1:**
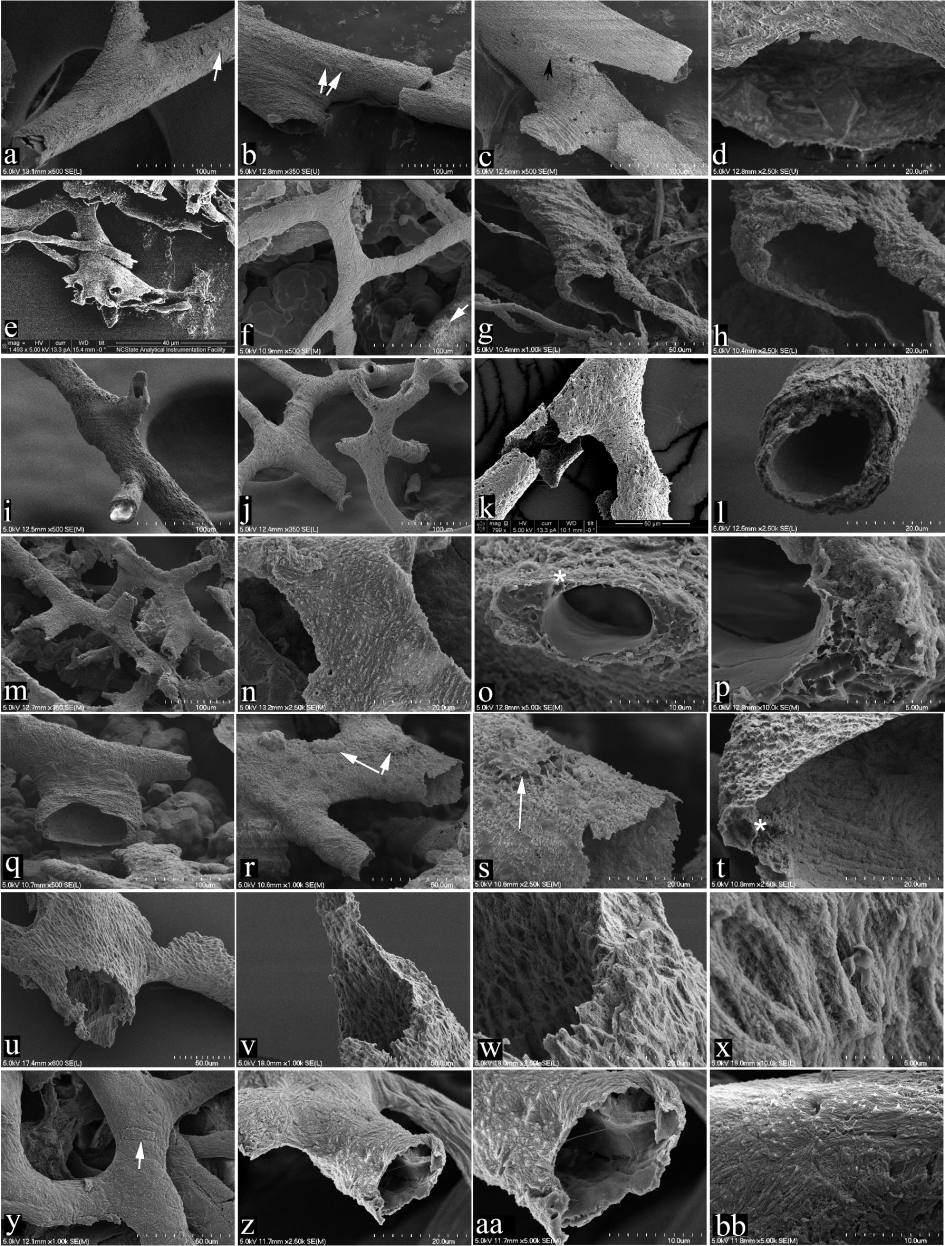
SEM images of vessels recovered from non-avian dinosaur and ostrich bone. **a**–**d**, MOR 2598, *B. canadensis*, **e**–**h**, MOR 10857, ceratopsian, **i**–**l**, MOR 555/USNM 555000, *T. rex*, **m**–**p**, MOR 1125, *T. rex*, **q**–**t**, MOR 1126, *T. rex*; **u**–**x**, MOR 1128, *T. rex*, **y**–**bb**, extant ostrich. Asterisks (*) denote accessory vessel, arrows mark apparent osteocytes on external vessel surfaces. Note that all vessels show a branching pattern and open lumen. Scale bars are as indicated.

SEM of vessels (Fig. 1a–bb) corroborated the hollow nature and integrity of the vessel walls from each dinosaur. Further, these vessels branch and taper, as in living vertebrates, and in some cases, gave rise to complexes of smaller vessels (Fig. 1e). The external textures in all but MOR 1128 (Fig. 1u–x) differ from the luminal surfaces (Fig. 1k; see also Figs. 22 and 23 in ref^122^). This pattern is also seen in ostrich vessels (Fig. 1z, aa, bb), and their characteristic striated texture is consistent with remnant collagen of the tunica externa. The striated, punctate surface seen in Fig. 1z, aa and bb of ostrich is virtually identical to the external texture of the dinosaur vessels in Fig. 1a, f, i, j, m, n and q. Osteocytes (Fig. 1a, b, r, s, arrows) could occasionally be seen on the vascular surfaces. Some vessels possessed small accessory vessels (Fig. 1o*, t,*). After isolation, vessels retained their three-dimensional character rather than collapsing after the mineral substrate was removed, consistent with the presence of an elastin-collagen complex^123^, secondary mineralisation, or a combination of these. MOR 1128 (Fig. 1u–x) was qualitatively different than the others, displaying much greater degradation and little heterogeneity of structure.

### TEM

**Fig. 2:**
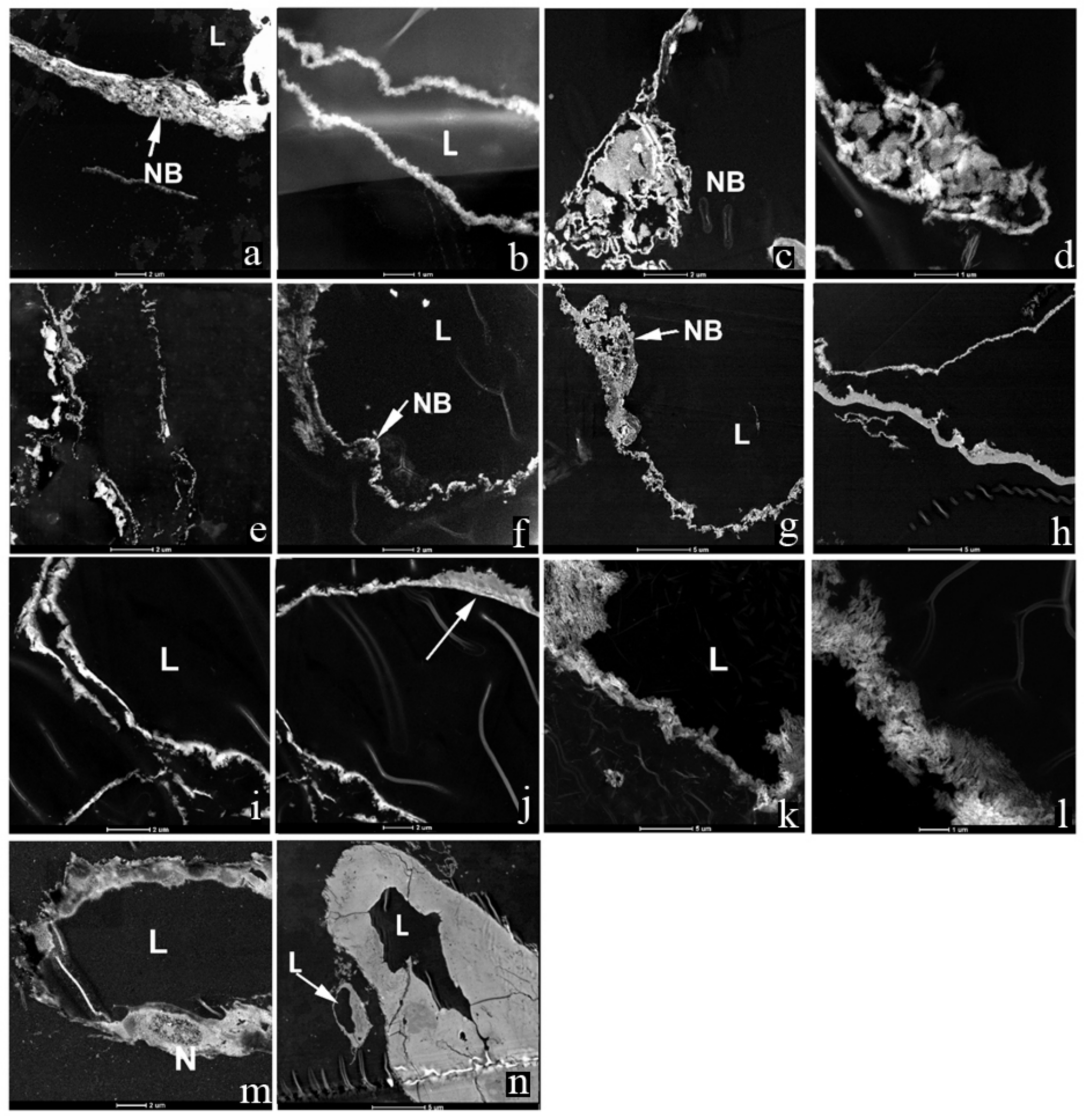
TEM images of **a,b**; MOR 2598; **c,d**; MOR 10857; **e,f**; MOR 555/USNM 555000; **g,h**; MOR 1125; **i,j**; MOR 1126; **k,l**; MOR 1128; **m,n**; *S. camelus* (ostrich). L, Lumen; NB, Nuclear bulge. Arrow in **j** shows fibers in the layer adjacent to lumen. Scale bar as indicated.

We used TEM to obtain high magnification, high resolution images of the vessel walls from each dinosaur specimen (Fig. 2a–l), and then compared these with corresponding vessels, osteocytes and extra cellular matrix (ECM) of extant ostrich bone (Fig. 2m, n). Micromorphology was similar to extant endothelia, and in some cases, a structure morphologically consistent with an endothelial ‘nuclear bulge’ (NB) could be seen extending into the lumen (e.g., Fig. 2a, c, d, g), as seen in extant ostrich vessels (Fig. 2m, n). Additionally, dinosaur vessels showed a complex wall architecture that varied in density, with apparent thinned cytoplasmic extensions. A distinct accumulation of fiber-like structures on the external surface of some vessel walls (Fig. 2j, arrow) is seen at high magnification, suggesting the presence of elastin fibers. Notably, the preservation differs in MOR 1128, with the vessels exhibiting a more crystalline morphology and lacking structural variation (Fig. 2k, l).

### nano-CT

**Fig. 3:**
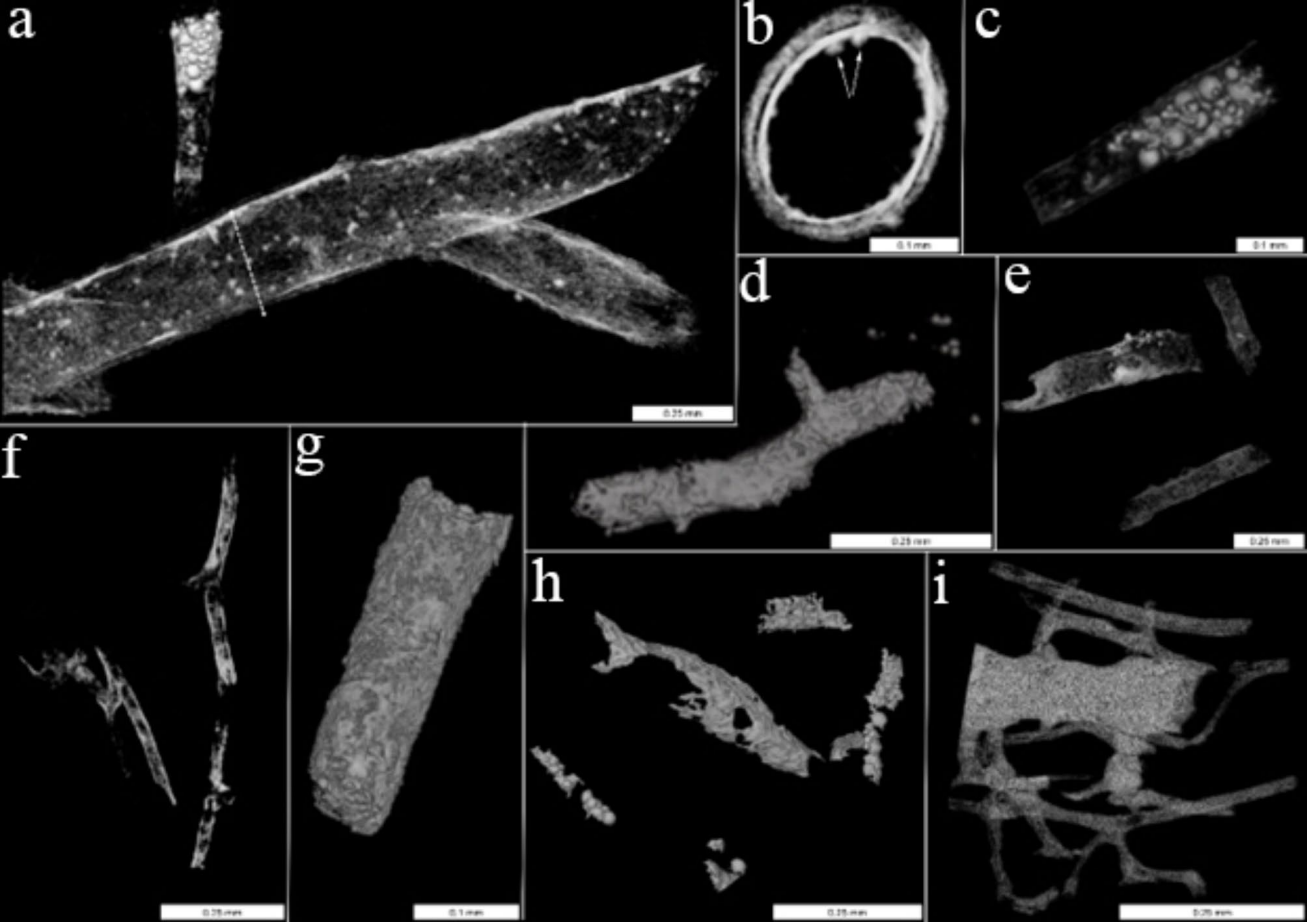
Representative nano-CT images of dinosaur vessels. **a**–**c**, MOR 2598. **a**, overview, shown with a smaller vessel containing abundant rounded spheres. **b**, Cross-section, taken at the level of dashed line in **a**. **c**, vascular inclusions from MOR 2598. **d**, MOR 10857 showing heterogeneous surface texture and branching of vessels; **e**, MOR 555/USNM 555000 shows complex vessel walls with varying density; **f**, MOR 1125 vessels are long, hollow and show branch points for smaller vessels; **g**, MOR 1126 has dense outer layer, perhaps indicating encrustation; **h**, MOR 1128 vessels are highly fractured, with vessel on lower left containing dense, spherical vascular inclusions; **i** Vessels liberated from extant ostrich (*S. camelus*) show greater interconnectivity and more branching of vascular network. Scale bars are as indicated.

To better visualise the composition and layering of the fossil-derived vessel walls in three dimensions, we employed high-resolution nano-CT to isolated vessels from each dinosaur specimen (Fig. 3a–i). nano-CT verified the complexity of most dinosaurian vessel walls, highlighting structural and density variation, which were most apparent in cross-section (Fig. 3b). The innermost layer was dense and continuous, consistent with endothelium and the accompanying basement membrane. However, the outer layers distal to the lumen are less dense, similar to the tunica adventitia of extant vessels^124–126^, which is comprised of dense and loose connective tissue layers (Supplementary Information Fig. S1 and^127^). Small, rounded structures (Fig. 3b, arrows) protrude into the lumen in some renderings, similar to the TEM data. In some cases, dense rounded structures (Fig. 3a, c) can be seen filling the vessel lumen. The ceratopsian vessels (MOR 10857; Fig. 3d) appear denser and less transparent; and were more similar to vessels from *T. rex* specimen MOR 1126 (Fig. 3g) than the rest of the specimens examined. Three vessels from MOR 555/USNM 555000 are shown in Fig. 3e. These longitudinal views show a dense and continuous inner layer bounding the lumen, with features arising from this layer that suggest variation in vessel wall density and thickness. MOR 1125 vessels (Fig. 3f) are long and branch, giving rise to smaller vessels. The lining is continuous and vessels are filled with material that is visually distinct from the vessel walls. Vessels from MOR 1126 (Fig. 3g) were histologically/morphologically similar to the ceratopsian vessels (Fig. 3d). Both appeared thick and dense, perhaps following encrustation with an iron-derived material as noted previously^41,80,128^. In MOR 1128 (Fig. 3h), the vessels were less well-preserved, more homogeneous, and more friable than in other specimens.

Ostrich vessels (Fig. 3i) shared features with dinosaur vessels; however, the walls were thicker, and they displayed more branching structures. The large central vessel was less transparent than the smaller ones, perhaps reflecting a thicker tunica media. Smaller ostrich vessels are similar in structure and density to most figured dinosaur vessels.

### Immunological and histochemical assays

**Fig. 4:**
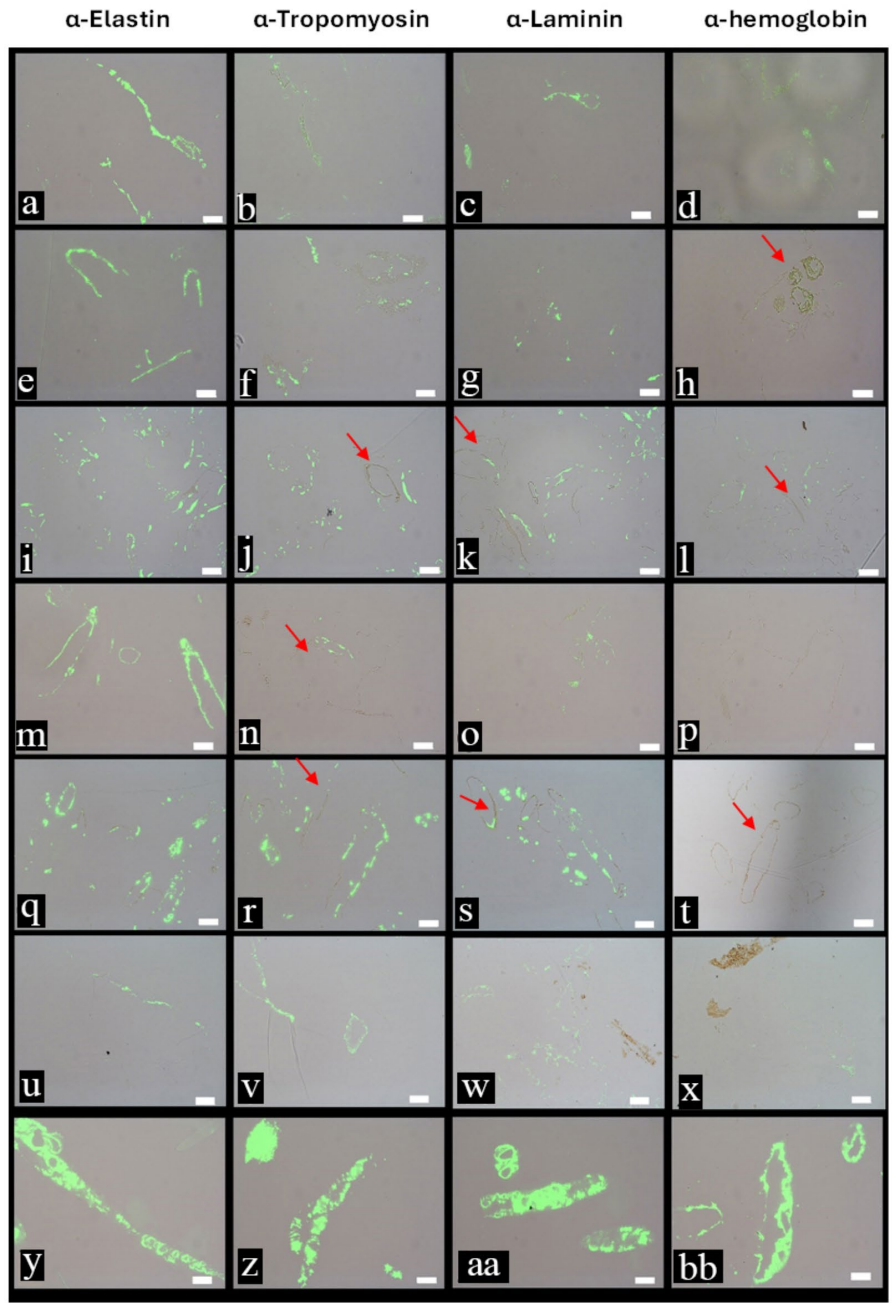
In situ immunohistochemistry (IHC) data taken in dual channels (transmitted light and FITC) where antibody–antigen complexes are visualized by fluorescence and localized to vessel walls. **a**–**d**, MOR 2598; **e**–**h**, MOR 10857; **i**–**l**, MOR 555/USNM 555000; **m**–**p**, MOR 1125; **q**–**t**, MOR 1126; **u**–**x** MOR 1128; **y**–**bb**, ostrich. Column 1, tissues exposed to elastin antibodies (see Supplementary Information for description and characterization); Column 2, exposed to antibodies to tropomyosin; Column 3, exposed to laminin antibodies, and Column 4, exposed to anti-ostrich hemoglobin antibodies. All fossil data collected under the same parameters, allowing direct comparison of binding strength/avidity. Arrows show regions of crystallinity. Scale bars 20 μm.

To ascertain the molecular composition of the vessel walls, we employed two immunological methods that localise antibody–antigen complexes to the vessels: immunohistochemistry (IHC, Fig. 4a-bb, Supplementary Information Figs. S3, S4) and immunogold labeling (IG, Supplementary Information Fig. S6). Figure 4 shows binding of antibodies against elastin (column 1), tropomyosin (column 2) and laminin (column 3)—all known components of vertebrate blood vessels—and against an avian hemoglobin (column 4), which may deposit on vessel walls after *post-mortem* erythrocyte lysis. All vessels show the most intense binding to the long-lived protein elastin; however, tropomyosin and laminin binding could also be seen.

Ostrich vessels reacted strongly to anti-ostrich hemoglobin antibodies (Fig. 4bb), but dinosaur vessels varied in hemoglobin reactivity. MOR 2598 and MOR 555/USNM 555,000 (Fig. 4d, l) showed some hemoglobin reactivity, but MOR1125 and MOR 1126 (Fig. 4p, t) show none. A dark brown, crystalline material, presumably an iron precipitate, was associated with some vessels (Fig. 4, red arrows) as previously reported^41,80,128^. Antibody binding was greatly diminished in the crystalline areas, but some binding was observed in a translucent region adjacent to the mineral layer.

To independently validate the presence of elastin (Fig. 4), we applied antibodies to desmosine, an amino acid intimately involved in elastin crosslinking, to extant vessels (Supplementary Information Fig. S3)^129^. Binding avidity varied among specimens, but in all cases, binding to ostrich vessels was much more intense, as expected.

Negative controls, consisting of (1) withholding primary antibody to control for spurious binding of secondary antibody or label; and (2) exposing vessels to antibodies against peptidoglycan, to control for the presence of bacteria, are shown in Supplementary Information Fig. S4. In a second set of negative controls, we exposed all vessels to lactophenol cotton blue (Supplementary Information Fig. S5a-i), which recognises bacterial and fungal polysaccharides^130,131^. We noted some highly localised staining in some ancient vessels associated with hyphae-like threads, for example, as seen in MOR 10857 (Supplementary Information Fig. S5b, arrows). Staining was limited to much smaller and/or septate structures within the larger vessels when visible. Most vessels, however, showed no reactivity.

Using the same suite of antibodies described above, we applied a secondary antibody tethered to small, electron-dense gold beads to detect binding in situ at higher resolution. Supplementary Information Fig. S6a-bb shows localisation of antibody–antigen (ab–ag) complexes in specific regions of vessel tissues. Only data for elastin antibodies are shown, because anti-elastin binding was greatest in other assays. Images were taken using brightfield (BF, Supplementary Information Fig. S6, first and third columns) and high angle annular dark-field (HAADF, Supplementary Information Fig. S6, second and fourth columns) in both lower and higher magnifications. At high magnifications, small electron-dense filaments can be seen on the external surface of MOR 2598 vessel wall (Supplementary Information Fig. S6c, arrows). Tagged elastin antibodies bind preferentially to this layer. Supplementary Information, Fig. S6e–h represents vessels recovered from MOR 10,857. Under high magnification (Supplementary Information Fig. S6g, h), beads align on only one side of the vessel wall, but binding is neither indiscriminate nor non-specific, and shows limited or no binding to other structures, supporting the presence of elastin epitopes and antibody specificity. The vessels from MOR 555/USNM 555000 (Supplementary Information Fig. S6i–l) show limited binding to these antibodies. These vessels are crystalline and electron dense, likely representing mineral precipitation on vessel walls, which we have previously shown to inhibit antibody binding^41,80,88,128^. Supplementary Information, Fig. S6m–p also shows electron dense regions (dark in BF, bright in HAADF) in vessels recovered from MOR 1125. Tagged antibodies localise to the less electron dense material but still show extreme selectivity to particular regions of the vessel wall. Similarly, MOR 1126 (Supplementary Information, Fig. S6q–t) and MOR 1128 (Supplementary Information Fig. S6u–x) show binding is limited to electron-lucent regions but very few beads are seen in association with crystalline portions. Mineral crystals can be seen in the lower left of Supplementary Information Fig. S6w, x, but no antibody binding is seen in that region. In all cases, antibody binding was less than in extant ostrich vessels (Supplementary Information, Fig. S6y–bb), consistent with binding patterns observed in IHC (Fig. 4).

### ToF-SIMS combined with FEG-SEM

We employed ToF-SIMS in combination with FEG-SEM imaging to independently investigate the presence of proteinaceous materials in the vessel-like structures. In all samples, peptides/proteins were detected by the presence of typical protein-related secondary ions (Supplementary Information, Fig. S7a–c). These N-containing fragment ions occur at high signal intensities in positive ion spectra of pure proteins and peptides^117–119^, and usually comprise the side group of a specific amino acid together with the C-N portion of the peptide backbone^117^. These same ions also occur in ToF-SIMS spectra of free amino acids but, importantly, free amino acids also generate comparably intense peaks corresponding to intact molecular ions^133^, which are notably lacking in spectra of peptides/proteins. Here, identification of peptides/proteins in the vessel samples is based on: (1) the combined observation and assignment of peaks in the fossil spectra to all major protein-associated N-containing fragment ions at high mass resolution (Supplementary Information, Fig. S7a); (2) comparable intensity distributions of these ions in the fossil spectra, consistent with peptides/proteins (Supplementary Information Fig. S7b, c); (3) a lack of peaks corresponding to intact free amino acids (Supplementary Information, Fig. S7d); and (4) an absence of other major N-containing organic fragments in the ToF-SIMS spectra.

**Fig. 5:**
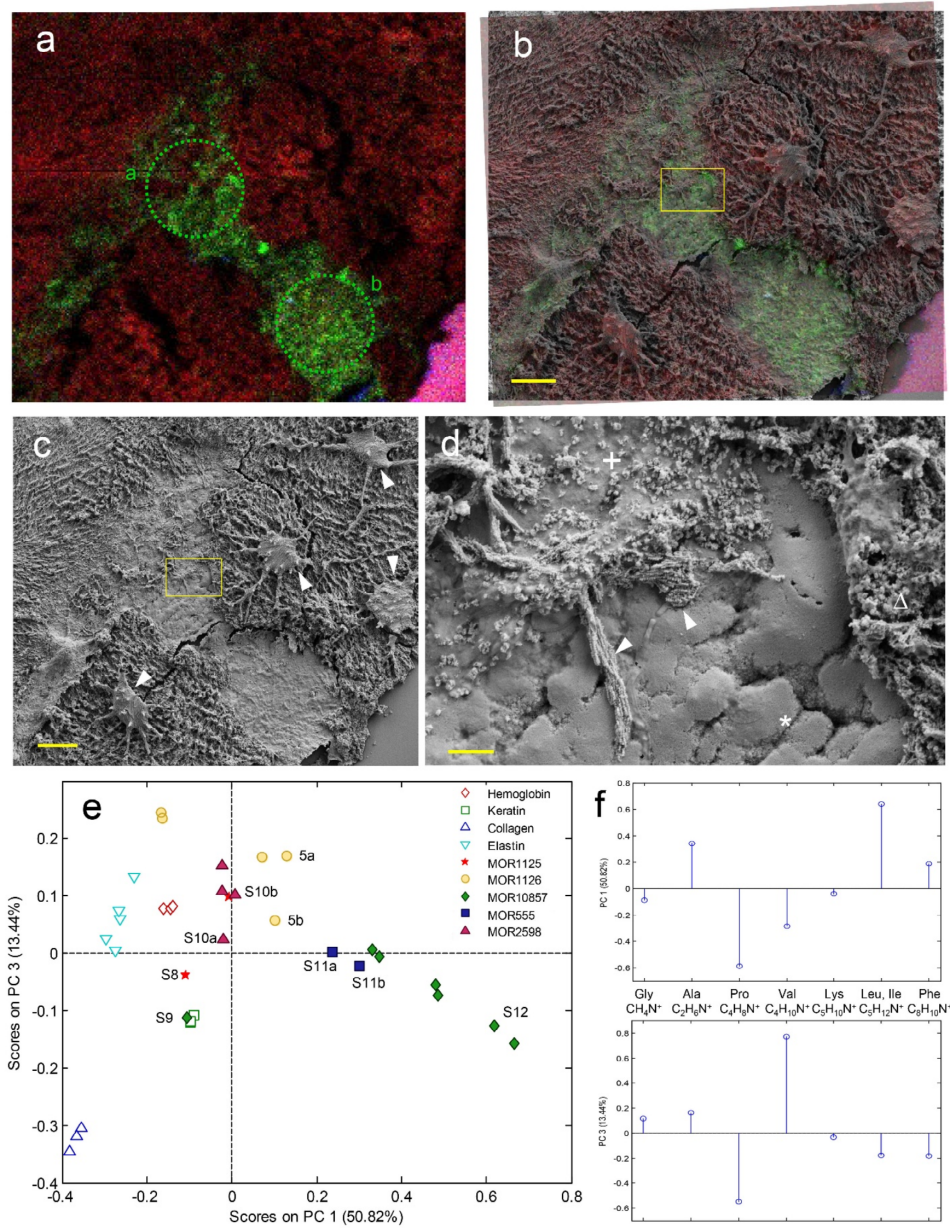
Detection of proteins in fossil vessels by ToF-SIMS. Overlay TOF-SIMS image of MOR 1126 (**a,b**) showing the added intensity of ions representing proteins in green and iron phosphate in red. (**b**) Superimposed ToF-SIMS image (as in (**a**)) and SEM micrograph of the same area on the vessel surface. (**c**) SEM image as in (**b**). Arrowheads indicate structures resembling osteocytes and the yellow box indicates the area magnified in (**d**). (**d**) Magnified SEM micrograph highlighting various microstructures: flat, protein-containing layer (*) located below a porous inorganic layer (Δ), porous inorganic layer infiltrated with structureless deposit (+), and filaments resembling collagen fibers (arrowheads). (**e**) Score plot from spectrum PCA that includes fragment ion intensities in positive ToF-SIMS spectra of four reference proteins and protein-rich ROIs on the fossil vessel surfaces. Labels of specific data points indicate spectra from ROIs displayed in Fig. 5a and Supplementary Information, Fig. S8–12, as marked by green dotted lines. (**f**) PC1 (upper) and PC3 (lower) loadings from PCA as in (**e**), for included protein fragment ions (associated amino acids are indicated). Scale bars are 10 μm in (**a**–**c**) and 1 μm in (**d**).

Protein-related fragment ions were observed in specific, sharply delineated regions on the fossil surfaces, which were otherwise dominated by iron- and phosphate-containing minerals (Fig. 5a–d and Supplementary Information Fig. S8–S12). In MOR 1126 (Fig. 5a–d), subsequent analysis by FEG-SEM revealed that the peptide/protein fragment ions originated from sheet-like matter that appeared to underlie a variably porous and fibrous layer (Fig. 5d, asterisk). The latter was dominated by iron/phosphate-containing minerals and showed morphological features (e.g., a ~ 68 nm banding pattern; Fig. 5d, arrowheads) consistent with collagen fibers and osteocyte-like cellular structures (Fig. 5c, arrowheads) that may indicate exogenous mineral deposition or secondary replacement of the originally organic materials. Protein fragment ions were detected in areas where the porous/fibrous layer had been fragmented or unintentionally removed during the sample preparation process, exposing the underlying sheet-like matter of the vessel tissue.

In addition to sheet-like material, protein signals were also observed in regions where an amorphous mass seemed to have infiltrated the iron/phosphate layer, possibly indicating organic deposits associated with microbial activity (Fig. 5d, ‘+’). Furthermore, structures morphologically consistent with fungal hyphae could be seen on the sheet-like surface adjacent to infiltrated areas of the iron/phosphate layer, consistent with histochemical staining patterns.

Similar observations were made in all fossil samples; i.e., peptide/protein detection in a sheet-like material located below a porous iron/phosphate-rich layer (Supplementary Information, Fig. S8a–d, S9a–d, S12a–d), as well as in various microstructures of presumed microbial origin (Supplementary Information, Fig. S10a–d, S11a–d). For example, in MOR 555/USNM 555000, peptides/proteins were identified in clusters of sub-spherical bodies displaying typical microbial characteristics (Supplementary Information, Fig. S11d), and MOR 2598 exhibited proteins localised to thread-like structures that were morphologically consistent with fungal hyphae (Supplementary Information, Fig. S10c, d). Notably, all samples also showed the presence of peptides/proteins in a sheet-like matter without any apparent association with either bacteria or fungi.

Furthermore, to investigate if the origins of the observed proteinaceous materials (endogenous *versus* microbial) are reflected in the ToF-SIMS data, spectra from protein-rich regions on the fossil surfaces were evaluated using principal component analysis (PCA). The PCA included signal intensities of seven major protein fragment ions in spectra from regions of interest (ROIs) of the fossil samples, together with spectra from four pure protein reference samples: hemoglobin, keratin, collagen, and elastin (Fig. 5e, f). The PC1–PC3 score plot showed that the four protein standards all occur in the left part of the plot. They are further separated from one another in chemospace, indicating significant differences between the ToF-SIMS spectra. Whereas some of the fossil spectra are located in the vicinity of the protein reference samples, most are placed on the right-hand side of the plot (i.e., higher PC 1 scores), which, according to the PC 1 loadings (Fig. 5f), correspond to higher relative intensities of C_2_H_6_N^+^ (Ala) and C_5_H_12_N^+^ (Leu, Ile), and lower intensities of C_4_H_8_N^+^ (Pro) and C_4_H_10_N^+^ (Val) relative to the modern protein references. The vertical scale in the score plot (PC 3) mainly corresponds to relative intensity differences between C_4_H_8_N^+^ (Pro) and C_4_H_10_N^+^ (Val), and is associated with spectral differences between the various protein references. The labelled data points in the score plot (Fig. 5e) correspond to spectra acquired from the microstructures indicated by dotted lines in Fig. 5a, b and Supplementary Information, Figs. S8a, b–S12a, b. Notably, the data points 5b, S8, S9, and S10a, which all are associated with structures without any apparent microbial origin, seem to be located in a region slightly closer to the protein references, as compared to 5a, S10b, S11a, and S12b, which derive from suspected microbial features. However, this apparent separation in chemospace is not without exceptions (e.g., data point S12), indicating that the spectral features included in the PCA analysis (i.e., the seven protein fragment ions) are insufficient to confidently distinguish endogenous proteinaceous matter from peptides/proteins of microbial origin.

## Discussion

Our comprehensive set of imaging, ultrastructural and molecular analyses detected three main components of vascular remains recovered from six Cretaceous non-avian dinosaurs (four tyrannosaurids, a ceratopsian and a hadrosaur, which were separated in time by at least 10 million years): endogenous organics, secondary replacement structures (mineral deposits) and invasive microorganisms (e.g., fungal-like hyphae, either ancient or modern). We were also able to eliminate modern bacterial biofilm (see^85^) as the primary source of the vessel-like structures because they: (1) were texturally distinct, with a luminal surface that differed from the fibrous exterior; (2) showed micromorphological similarity to extant analogues at multiple levels of visualisation; (3) exhibited chemical sequestration (as revealed by color and textural differences from the surrounding matrix at the sub-micron scale); (4) were hollow and maintained their shape after demineralisation; and (5) displayed distinct and highly specific binding patterns when exposed to relevant antibodies; all characteristics not found in bacterial structures (e.g., biofilm)^113^.

Even though we detected microorganismal components in the samples, our larger sample set shows that the bone analysed by Saitta et al.^91^ is not representative for drawing more general conclusions regarding the retention of endogenous molecules in the fossil record. Furthermore, invasion of fossil bone by either fungi or bacteria strongly supports the presence of organics within the bone; microbes do not grow without an organic source. Finally, two of the dinosaurs used in the present study previously yielded protein sequence data that were consistent with vertebrates, not microbes^14^.

Likewise, by incorporating fossils from both fluvial sandstones and mudstones, we tested the extent to which the depositional environment acts as a predictor of vascular preservation. Vessels were recovered from each dinosaur specimen analysed in this study, although these varied in fidelity at both the morphological (Figs. 1, 2 and 3 and Supplementary Information, Fig. S2) and molecular (Fig. 4 and Supplementary Information, Figs. S3, S6, S7) levels. Hence, highly specific burial conditions do not appear to be a requirement for at least the structural integrity of vessels to be maintained across deep time. However, while the often pristine morphology revealed structural heterogeneity in the vessel walls, these were predominantly secondarily mineralised, as were the surrounding osteocytes and collagen fiber bundles. Importantly, though, the inorganics were always associated with an organic complement showing characteristics of the original vessel wall, and suggest that the ancient vascular remains indeed represent fossilised blood vessels (*contra*^85,91^). The data presented herein thus support the hypothesis that the preservation of endogenous organic structures can be enhanced by association with bone mineral^81^.

Phylogenetic relationships and evolutionary processes can potentially be explored at the molecular level in fossils, and have recently been attempted by examining dinosaur hard tissues using Raman spectroscopy^55,93,134,135^. The scientific approach employed by Wiemann and colleagues builds on the assumption that ancient organic matter (including all proteins) have been transformed into advanced glycoxidation and lipoxidation end-products; these retain original molecular signals that can be measured using Raman spectroscopy, in turn allowing inferences on the phylogenetic, metabolic and/or physiological status of extinct organisms. However, concerns have been raised regarding the authenticity of the acquired spectra^136^ (see also^137^), and whether these represent actual fossil signatures or instrumental artefacts^138^. Likewise, the interpretation of some of the data has been called into question^139^ (see also^140^), and to further compound these issues, PCA was employed by both Wiemann et al.^55,93,135^ and Norell et al.^134^ to support the endogeneity of the acquired spectra, despite the risk of inadvertently introducing artefacts that could potentially skew that data in favor of a desired interpretation^141^.

The results of our extensive investigation add yet another level of uncertainty, as the original organics were always associated with a microbial complement, which unquestionably would affect any Raman investigation (as it relies on bulk measurements). This is particularly noteworthy as our samples derive from the same, or directly comparable, formations and rock units as the fossils analysed by Wiemann et al.^55,93,140^. Accordingly, we encourage the use of multiple complementary analytical techniques when examining deep-time fossil materials, and argue caution when inferring aspects of, e.g., phylogeny and physiology solely from Raman data, or indeed, any single technique.

## Conclusions

We tested three hypotheses in the present work: (1) the vessels are endogenous to the dinosaurs from which they derive; (2) the depositional environment is a predictor of vascular preservation; and (3) vascular integrity in fossils is not dependent upon geological age or taxon. Evidence for endogeneity of the vascular structures in non-avian dinosaur bone includes morphology using multiple techniques (LM, SEM, TEM, and nano-CT) and resolutions, the presence of anastomoses, the continuity of vascular wall structures, and in situ antibody binding. In addition, the combined ToF-SIMS and SEM investigation showed that peptide/protein signals originate from specific structures on the fossil surfaces, including: (1) a sheet-like layer positioned immediately below a porous substrate of mineralised collagen fibers; and (2) structures with obvious microbial origin. The depositional environment did not seem to be a factor in the ability to retrieve vessels from bone since our specimens derive from both sandstones and mudstones. However, the preservation was less immaculate in one sample obtained from mudstone. The study specimens included ornithischian (ceratopsian and hadrosaurian) and saurischian (*T. rex*) taxa, and were separated in time by ~ 10 million years, indicating that neither taxonomy, time, nor depositional setting exclusively influence this type of preservation. The microbial component we detected by ToF-SIMS and SEM could have arisen from coeval invasion, or be more recent, either when the sediments were close to the surface or after removal from the stabilising burial environment. It has been shown that microbes can enhance preservation^142,143^, and this possibility should be further explored when dealing with ancient remains.

## Electronic supplementary material

Below is the link to the electronic supplementary material.

Supplementary Material 1

## Data Availability

All data are included in the main text or supplemental information except extensive controls; these data are available upon request to MHS.
