# Supplementary material for: Taphonomic variation in vascular remains from Mesozoic non-avian dinosaurs
Source: Sci Rep. 2025 Feb 5;15:4359. doi: 10.1038/s41598-025-85497-y (PMC11799182; doi:10.1038/s41598-025-85497-y)
Supplement: Supplementary file 1 — Supplementary Material 1 [file 41598_2025_85497_MOESM1_ESM.docx]

**Supplementary information:**

**Taphonomic variation in vascular remains from Mesozoic non-avian**

**dinosaurs**

Schweitzer MH, Zheng W, Dickinson E, Scannella J, Hartstone-Rose A, Sjövall P, Lindgren J

**Materials and methods**

# **Blood vessel collection**

Cortical bone fragments from four specimens of *Tyrannosaurus rex* (MOR 555/USNM 555000), MOR 1125, MOR 1126, MOR 1128 but see (*1*)), *Brachylophosaurus canadensis* (MOR 2598), and an indeterminate ceratopsid (MOR 10857) (Table S1) were collected before consolidants were applied. All except MOR 555/USNM555000 were sampled either during excavation, or directly from jacketed specimens upon opening. MOR 555 was collected in 1991, but fragments were reserved without consolidants during preparation of the specimen and stored in sealed glass jars or at -80C. All bone fragments were demineralized in ethylenediaminetetraacetic acid (EDTA) (500 mM, pH 8.0) for at least 2 weeks with daily buffer changes. Vessels were collected and washed with e-pure water to remove residual EDTA for LM imaging and further treatment.

Table S1. Dinosaur specimens used in this study.

| **Specimen no.** | **Taxon** | **Geologic Formation** | **Lithology** | **Geography** |
| --- | --- | --- | --- | --- |
| MOR  555/USNM  555000 | *Tyrannosaurus rex* | Hell Creek Formation (upper) | sandstone | McCone Co., MT |
| MOR 1125 | *Tyrannosaurus rex* | Hell Creek Formation (lower) | sandstone | Garfield Co., MT |
| MOR 1126 | *Tyrannosaurus rex* | Hell Creek Formation (lower) | mudstone | Garfield Co., MT |
| MOR 1128 | *Tyrannosaurus rex* | Hell Creek Formation (lower) | mudstone | Garfield Co., MT |
| MOR 2598 | *Brachylophosaurus canadensis* | Judith River Formation | sandstone | Phillips Co., MT |
| MOR 10857 | Ceratopsia indet. | Judith River Formation | sandstone | Valley Co., MT |

Small fragments of ostrich long bone (excised 9/26/2004), were incubated with 10% Zout (Dial Corp.) to defat, then stored under desiccation with an antifungal powder for approximately 17 years. These fragments were demineralized with 0.5 M EDTA pH 8.0 until most of the mineral phase was removed and remaining tissues were pliable. Demineralized bone was cut into 0.5–1 mm slices with a sterile razor blade, then washed with e-pure water ~10 times to completely remove EDTA. Bone slices were then digested with 1 mg/mL collagenase A (Roche10103578001) in Dulbecco's phosphate-buffered saline (D-PBS) pH 7.2 (with 0.1 g/L calcium chloride and 0.1g/L magnesium chloride added) at 37^o^C overnight or until the collagen matrix was completely removed. The remaining vessels were collected under magnification and washed with e-pure water to remove residual collagenase, then incubated for 1 hr at room temperature in neutral buffered 10% formalin for embedding.

***Microscopy***

**Transmitted light microscopy (LM)**

Vessels were examined with Zeiss Stemi-2000-C or Zeiss Axioskop 2 plus biological microscope; and captured using an AxioCam MRc 5 (Zeiss) in the Axiovision software package (version 4.7.0.0).

# **Scanning Electron Microscopy (SEM)**

Ancient vessels and 10% formalin fixed ostrich vessels were dehydrated with a graded ethanol series. Then, the specimens were transported to the Chapel Hill Analytical and Nanofabrication Laboratory (CHANL) core facility where they were subsequently critical point dried (Tousimis Semidri PVT-3) and sputter coated (Cressington 108 Auto) with ~70 angstroms of palladium gold. Prepared samples were then analyzed using the CHANL Hitachi S-4700 Cold Cathode Field Emission Scanning Electron Microscope at an accelerating voltage of 5kEV.

In addition to vessels recovered as above, some *Tyrannosaurus rex* (MOR 1125) vessels used in this study were collected and lyophilized in 2006 and stored at -90^o^C until use. They were brought to room temperature in PBS, mounted on stubs with double-sided carbon tape and also analyzed with SEM as above.

# **Transmission Electron Microscopy (TEM)**

Isolated *Tyrannosaurus rex* (MOR 1125, MOR 555, MOR 1126, MOR 1128), *Brachylophosaurus canadensis* (MOR 2598), and ceratopsid (MOR 10857) vessels were incubated in pyridoxal isonicotinoyl hydrazone (PIH) solution (5mM PIH in 50mM NaOH (*2*)) overnight at room temperature to chelate excess iron from the recovered tissues, then washed with e-pure water. PIH treated ancient vessels and 10% formalin fixed ostrich vessels were first embedded in 3% agar to stabilize the tissues, followed by embedding in LR White resin blocks after partial dehydration in 70% ethanol and infiltration with undiluted LR White water permeable embedding medium as previously described (e.g. ^1,2^). 90 nm sections were cut on a Leica EM UC6 Ultramicrotome, mounted on carbon-coated nickel grids (EMS Cat CFT200-NI), and stained with 5% methanolic uranyl acetate for 5 minutes and Reynold’s lead citrate for 8 minutes, then observed using the Talos F200X G2 electron microscope at the Analytical Instrumentation Facility (AIF) of North Carolina State University.

# **Nano computed tomography (CT)**

Because ostrich vessels are not mineralized in life, they are difficult to visualize without staining.

Therefore, we stained these extant vessels with an aqueous 2.5% I_2_KI solution (I_2_KI: one part iodine to two parts potassium iodide in an aqueous solution) for 2 x 72 hours. To produce a master solution of 5% I2KI, we first dissolved 10% w/v potassium iodide into e-pure water until clear, then added 5% w/v elemental iodine into the same solution. We then diluted the master solution to 2.5% I2KI with pure water.

Ancient vessels collected as above and stained ostrich vessels were embedded and stabilized with

3% agar in 1.5ml centrifuge tubes, scanned at a voltage of 80 kV and a power of 7w with Zeiss Xradia 510 Versa 3D X-ray Tomography System in the Analytical Imaging Facility (AIF) of North Carolina State University.

# ***IMMUNOHISTOCHEMISTRY***

## **Immunofluorescence**

200 nm sections of LR white embedded vessels, prepared as described above, were taken on a

Leica EM UC6 Ultramicrotome and dried overnight at 45^o^C to each well of a Teflon coated slide (Electron Microscopy Sciences). Antigen retrieval and quenching of autofluorescence were accomplished by etching with Proteinase K (PCR grade, Roche, 25 ug/ml) in 1X phosphate buffered saline (PBS) at 37˚C for 15 minutes, incubated in 500 mM EDTA pH 8.0 for 30 minutes, followed by two incubations in 1 mg/ml sodium borohydride (NaBH_4_) for 10 minutes each. Incubations were interrupted by two five-minute washes in PBS. Spurious binding was inhibited by 4% normal goat serum (NGS) applied to sections for 2 hours at room temperature. Sections were incubated overnight at 4^o^C in either primary antibody (Table S2A) diluted to final concentration in primary dilution buffer, consisting of 1% bovine serum albumin (BSA) (Fisher, BP1660-100), 0.1 % cold fish skin gelatin (Sigma G7765), 0.05% sodium azide (Sigma S-8032), and 0.01M PBS pH 7.2), or in primary dilution buffer without added antibodies to control for non-specific binding of secondary antibody. Anti-elastin and anti-desmosine antibodies were also diluted to 1:75 and incubated with 16 mg of elastin or 10mg desmosine (Table S2B) to inhibit binding by blocking the antibody binding site. These blocked antibodies were then applied to sections as above, to test the specificity of those antibodies. All sections, including controls, were incubated for 2 hours in secondary antibody (biotinylated goat anti-rabbit IgG(H+L) (Vector BA-1000) diluted 1:500 for rabbit primary antibodies, biotinylated goat anti-mouse IgG (H+L) (Vector BA-9200), diluted 1:500 for monoclonal mouse anti-peptidoglycan, Sections were then incubated with Fluorescein Avidin D (FITC, Vector Laboratories A-2001) for 1 hour at RT. All incubations were interrupted by sequential washes (2 times for 10 minutes each) in PBS w/Tween 20, followed by two 10-minute rinses in PBS. Finally, sections were mounted with Vectashield H-1000 mounting media, and coverslips were applied. Sections were examined with Zeiss Axioskop 2 plus biological microscope and captured using an AxioCam MRc 5 (Zeiss) with 10x ocular magnification on the Axioskop 2 plus in the Axiovision software package (version 4.7.0.0).

**Table S2A**. Primary antibodies with dilutions used in Immunofluorescence

| **Antigen** | **Antigen Species** | **Antibody Type** | **Host** | **Final Dilution** | **Source** |
| --- | --- | --- | --- | --- | --- |
| **Laminin** | Purified laminin from  Engelbreth-Holm-Swarn  (EHS) murine sarcoma | Polyclonal | Rabbit | 1:75 | BioRad AHP420 |
| **Actin** | Chicken | Polyclonal | Rabbit | 1:75 | Capralogics, Inc.  P00851 |
| **Tropomyosin** | Chicken | Polyclonal |  | 1:50 | Abcam ab11190 |
| **Hemoglobin** | Ostrich | Polyclonal | Rabbit | 1:75 | Genscript  70594 |
| **Bovine Elastin** | Bovine | Polyclonal | Rabbit | 1:75 | Courtesy of R.  Mecham |
| **EPC Elastin** | Elastins pooled from pig, human, dog, chicken, rat and cow | Polyclonal | Rabbit | 1:75 | Elastin Products  Company,  Inc(EPC). TP592 |
| **Desmosine** | Desmosine covalently inked to keyhole limpets hemocyanin | Polyclonal | Rabbit | 1:75 | Elastin Products Company, Inc. DA878 |
| **Peptidoglycan** | *Streptococcus mutans* BHT cells | Monoclonal | Mouse | 1:75 | BioRad 7263-  1006 |

**Table S2B**: Protein and peptides used for antibody inhibition.

| **Protein and peptides** | **Species** | **Source** | **Concentration** |
| --- | --- | --- | --- |
| Elastin Peptides | Purified from bovine neck ligament | EPC CB573 | 16 mg/mL |
| Desmosine and  Isodesmosine (Equimolar  Mixture D866 and D975) | hydrolyzed bovine neck ligament | EPC MD687 | 10 mg/mL |

## **Immunogold Labeling (IG)**

Ancient vessels and extant ostrich vessels were also subjected to immunogold (IG) staining to demonstrate antibody-antigen complexes on tissues at higher resolution. 90nm sections of LR white embedded vessels (described above) were collected on carbon-coated nickel grids (EMS Cat CFT200-NI), incubated on droplets of PBS-Tween 20 for 10 minutes. 5% Normal Donkey serum (NDS) was applied to occupy non-specific binding sites and prevent spurious binding for 1 hour at room temperature. Sections on grids were then incubated with primary antibody, diluted 1:10 in primary dilution buffer as described, for 3 hour at room temperature. Sections were rinsed with TBS-Tween for 10 × 2 min. All grids were then incubated with secondary antibodies (12 nm Colloidal Gold AffiniPure Donkey Anti-Rabbit IgG (H+L) 1:20 (Jackson Immuno Research Inc Cat 715-205152) in secondary dilution buffer for 1 hr. Grids were then rinsed with PBS-Tween20 for 10x2 minute, followed by E-pure water rinses 3x30 seconds and dried with filter paper. Sections were stained with 5% methanolic uranyl acetate for 5 minutes and Reynold’s lead citrate for 8 minutes, then observed using the Talos F200X G2 electron microscope in AIF of North Carolina State University.

**Lactophenol cotton blue (LPCB) staining**.

Ancient vessels and several samples of pond fungi/biofilm were stained with LPCB. A drop of Lactophenol Cotton Blue Solution (Sigma 61335) was placed on a glass slide. Vessels from each dinosaur, isolated and recovered as above, were transferred to the stain droplet. The samples were then covered with a coverslip. After ~5 minutes, slides were examined with Zeiss Axioskop 2 plus biological microscope and images were captured using an AxioCam MRc 5 (Zeiss) with 10x ocular magnification on the Axioskop 2 plus in the Axiovision software package (version 4.7.0.0).

## **Propidium Iodide (PI) staining**

*Brachylophosaurus canadensis* (MOR 2598), *Tyrannosaurus rex* (Mor555/USNM 555000), MOR 1125, MOR 1126, MOR 1128 and MOR 10857 vessels, treated with PIH, and fixed ostrich vessels were incubated with 200 μl of 0.5% Triton X100 in PBS for 10 minutes, then washed with 200 μl PBS. All vessels (ancient and modern) were incubated with 40 μl propidium iodide (PI) solution (BD Biosciences 556463) for 30 minutes at room temperature in the dark. To remove excess stain, each sample was washed twice with 200 μl PBS. Cells were then transferred to 6-well "PTFE" Printed Slides, and Vectashield H-1000 Mounting medium and coverslips were applied. Images were obtained in the dark using a Zeiss AxioSkop2 Plus fluorescence microscope.

**Supplemental figures**


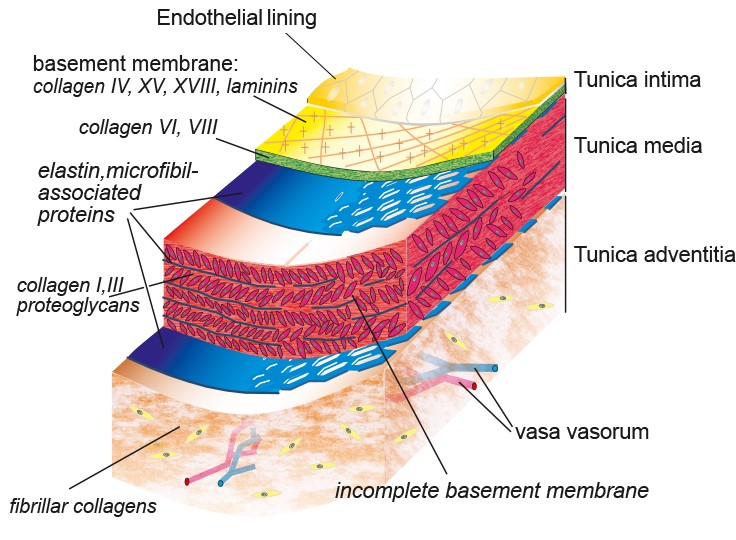


**Fig S1.** Schematic drawing showing the various layers of the heterogeneous blood vessel wall of living vertebrates, and some of the proteins related to these different regions. Modified from Eble and Niland (2009, figure 1^3^), and used with permission.

**
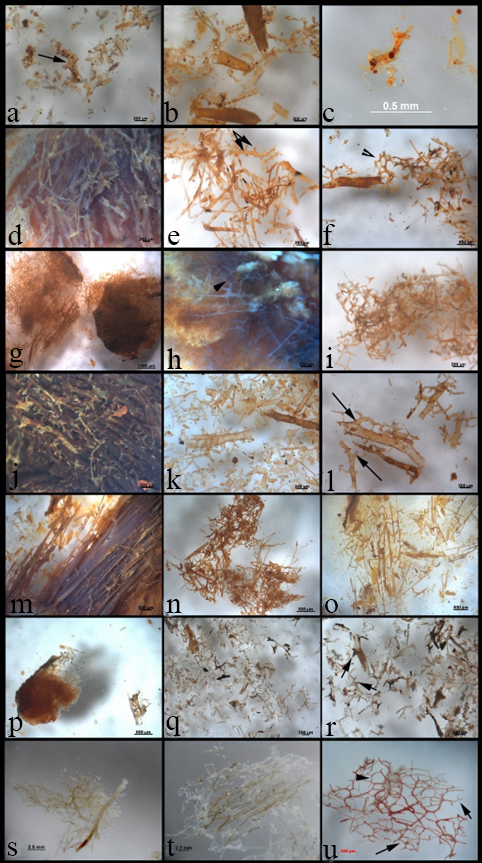
Fig. S2**. Transmitted light microscopy of MOR 2598, *Brachylophosaurus canadensis* (**a-c**); MOR 10857, unidentified ceratopsian (**d-f**); MOR 555/ USNM 555000, *Tyrannosaurus rex,* (**g-i**); MOR 1125, *T. rex*, (**j-l**), MOR 1126, *T. rex* (**m-o**); and MOR 1128, *T. rex* (**p-r**), compared with extant ostrich vessels (**s-u**), liberated from bone. Arrows indicate branching points, arrowheads show anastomoses.

Scale bars as indicated.


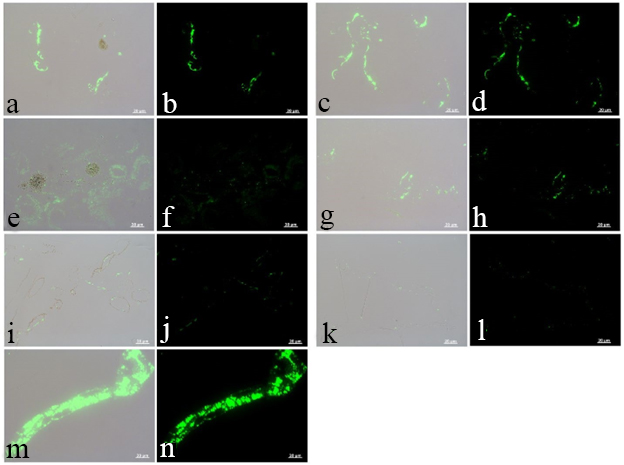


**Fig. S3**. Dinosaur vessels exposed to antibodies raised against desmosine (**a-l**) show positive, localized reactivity to vessel walls, but with far less intensity than in extant ostrich (**m,n**). **a-l** taken at 100ms, ostrich at 50ms. Fluorescent signal is limited to vessel walls with no binding elsewhere, and is stronger in transparent vessel regions than brown, crystalline areas. Scale bars as indicated.


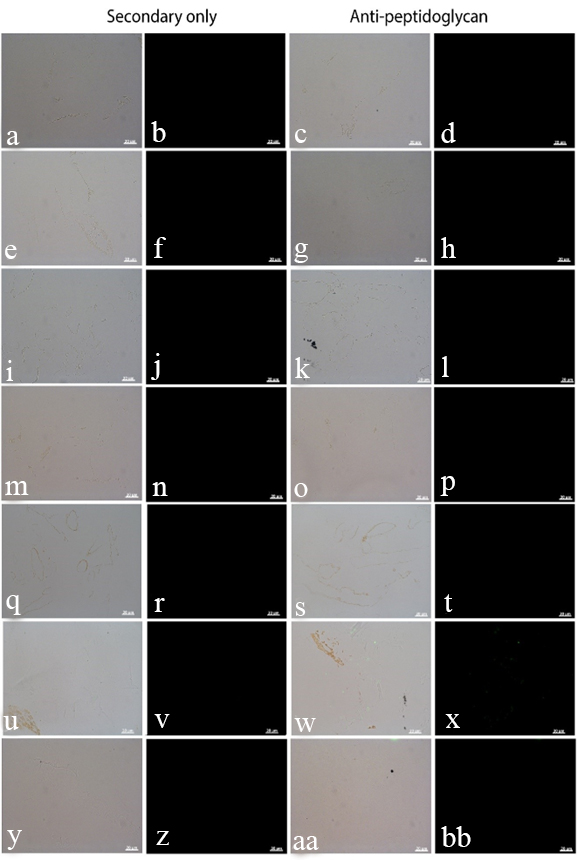


**Fig. S4.** Negative controls for IHC experiments. **a-d**, MOR 2598; **e-h**, MOR 10857; **i-l**, MOR555/ USNM555000; **m-p**, MOR 1125; **q-t**, MOR 1126; **u-x**, MOR 1128; **y-bb**, Ostrich. Column 1 and 3, brightfield imaging with fluorescent overlay, Column 2 and 4, FITC fluorescent filter. Column 1 and 2 exposed to no primary antibody, all other steps identical to Figure 4; Column 3 and 4, exposed to antibodies to bacterial peptidoglycan. Scale bars as indicated.


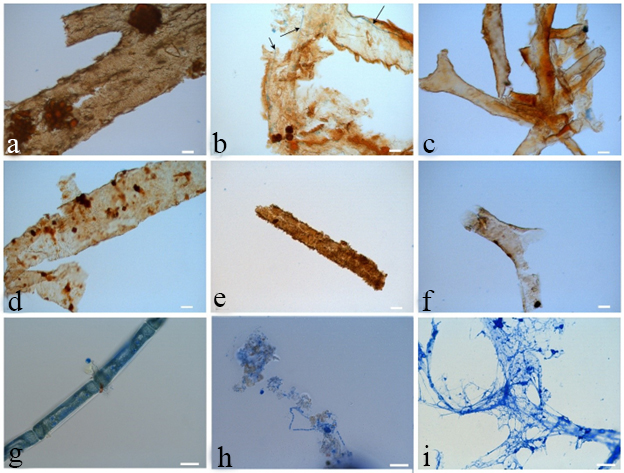


**Fig. S5**. Dinosaur vessels and extant fungi, stained with lactophenol cotton blue, a histochemical stain with strong affinity for fungal wall components. **a**, MOR 2598*, B*. *canadensis*; **b**, MOR 10857 (arrows show stained hyphae within vessel), ceratopsian; **c**, MOR 555, *T. rex*; **d**, MOR 1125, *T. rex*; **e**, MOR 1126, *T*. *rex*; **f**, MOR 1128, *T*. *rex*; **g-i**, samples of fungi and/or biofilm taken from recent pond. Scale bars, 20 µm.

**
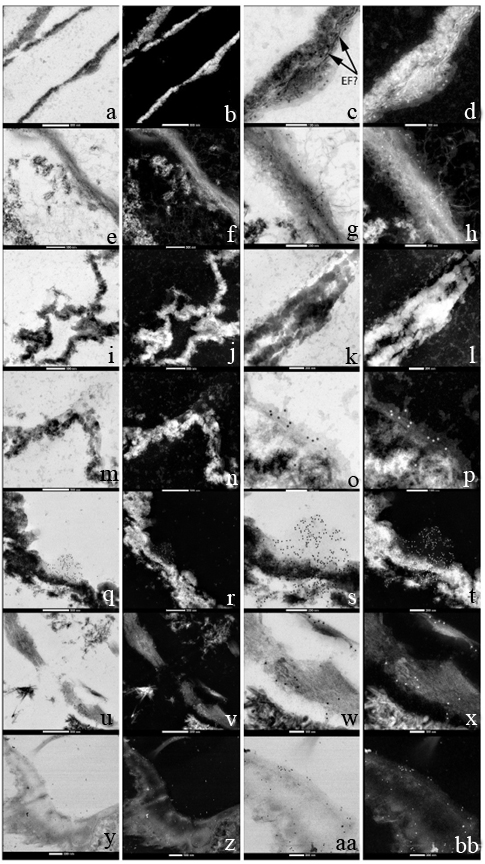
Fig. S6**. High resolution immunogold localization of antibody–antigen complexes using antibodies against elastin, visualized in brightfield (BF; columns 1 and 3) and high-angle annular dark-field imaging (HAADF; columns 2 and 4). **a-d**, low and higher magnification of MOR 2598 vessels. **e-h**, MOR 10857; **i-l**, MOR 555/USNM 555000; **m-p**, MOR 1125; **q-t**, MOR 1126; **u-x**, MOR 1128, **y-bb**, *S. camelus*. Antibody–antigen complexes are visualized as electron dense beads on vessel wall surfaces. Arrows show possible elastin filaments (EF). Scale bar for column 1 and 2 is 500 nm, column 3 and 4 is 200nm, except o p, w, x, scale bar is 100 nm.


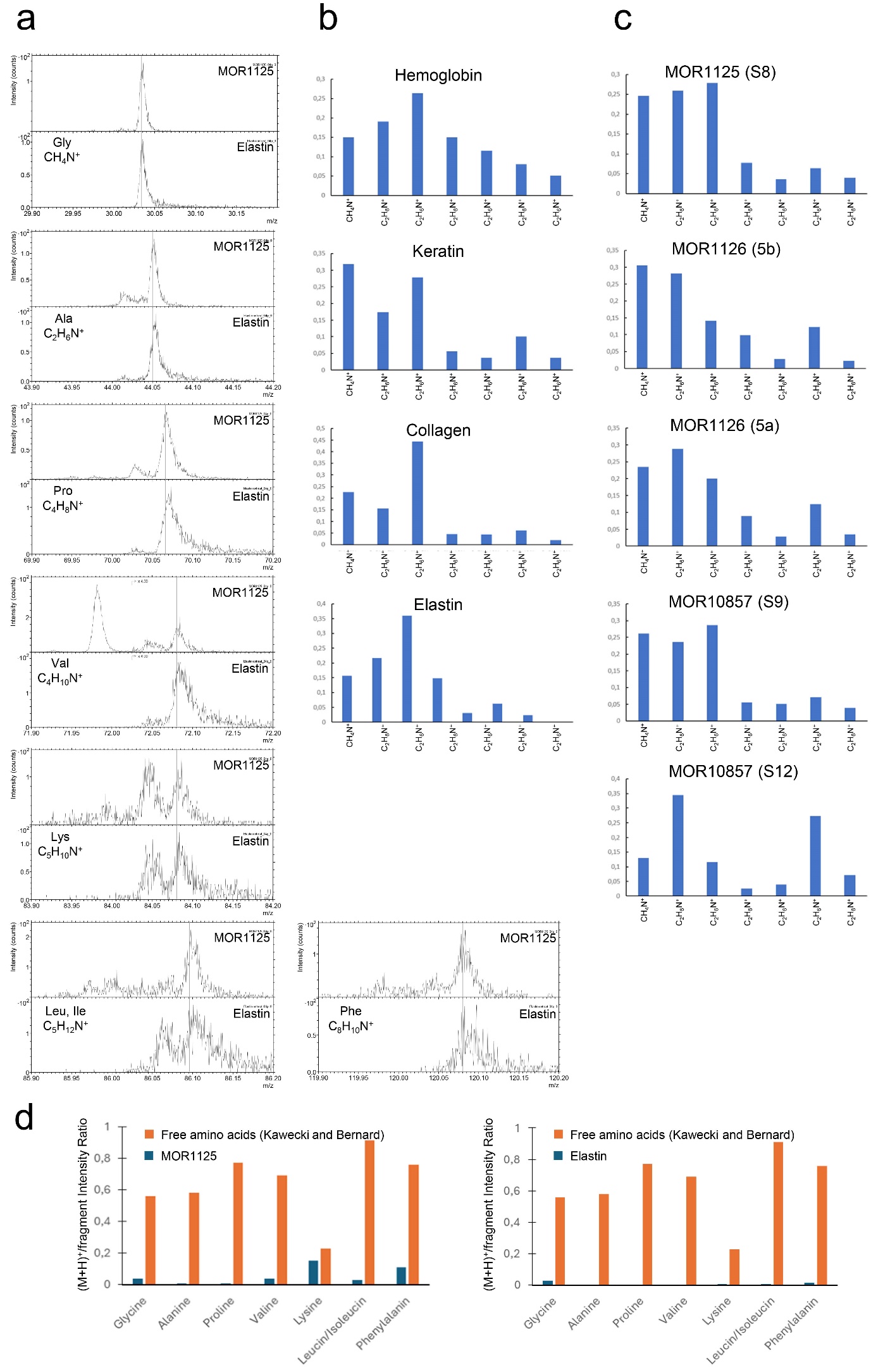


**Fig. S7**. Protein fragment ion detection and intensity distributions in reference proteins and fossil vessels. **a**, ToF-SIMS spectra of major protein fragment ions; comparison between fossil vessel (MOR1125, area S8) and a reference protein (elastin). The grey lines indicate theoretical *m/z* values for each fragment ion. The relatively low intensities (counts) of the protein fragment ion peaks are primarily due to the small areas of the ROIs from which the spectra are extracted. However, the low background noise level in the spectrum still results in a high signal-to-noise ratio, thus allowing for reliable detection and assignments of these peaks. **b**, Normalized intensity distributions of protein fragment ions for the reference proteins, and **c**, corresponding intensity distributions for selected fossil ROIs (specified in parentheses, referring to data points in Fig. 5e and areas indicated in the respective figures). The intensities were normalized to the sum intensity of all included ions (i.e., the sum of all intensities in each diagram is 1). **d**, Intensity ratio between the molecular ion and the protein fragment ion for the indicated amino acids, as estimated from Kawecki and Bernard (^4^) for free amino acids and as measured for MOR1125 (left) and for the elastin reference (right). No significant peaks were observed at the correct *m/z* values for the molecular ions in the fossil or elastin spectra, the non-zero intensities displayed for some of the amino acids are caused by overlap with adjacent peaks.


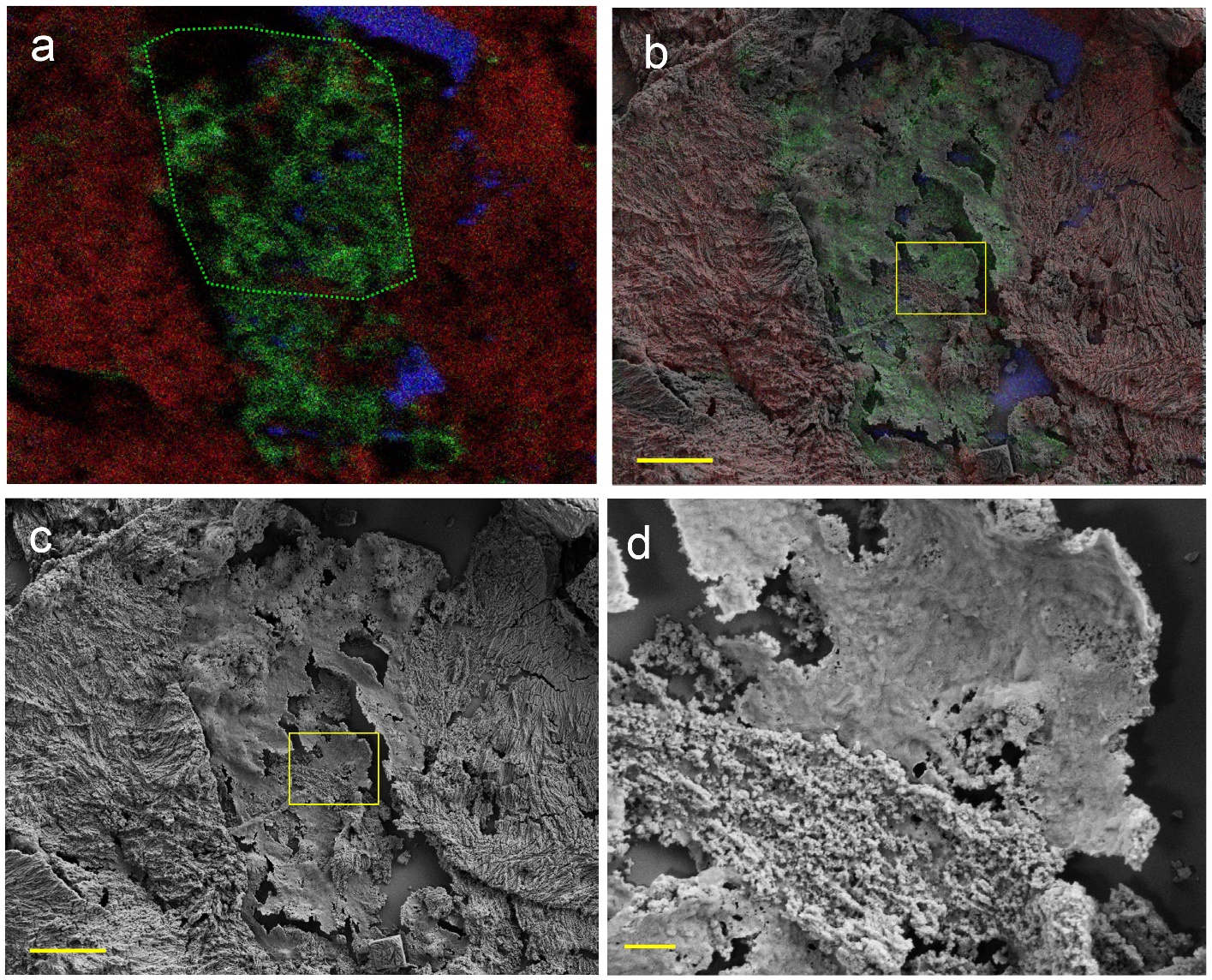


**Fig. S8**. Localization of detected proteins in vessel structure of MOR1125. **a**, Overlay ToF-SIMS ion image of ions representing proteins (green), iron phosphate (red), and silicon oxide (blue). The dotted green line indicates approximate area from which the spectrum used for intensity distribution analysis in Figure 5e, f was extracted (labelled S8). **b**, Superimposed ToF-SIMS image (as in **a**) and SEM micrograph of the same area on the vessel surface. **c**, SEM image as in **b**, where the yellow box indicates the area magnified in **d**. **d**, Magnified SEM micrograph of area generating protein fragment ions (flat structure, upper half) and iron oxide/phosphate-associated ions (porous material). Scale bars are 10 µm (**b-c);** 1 µm (**d)**.


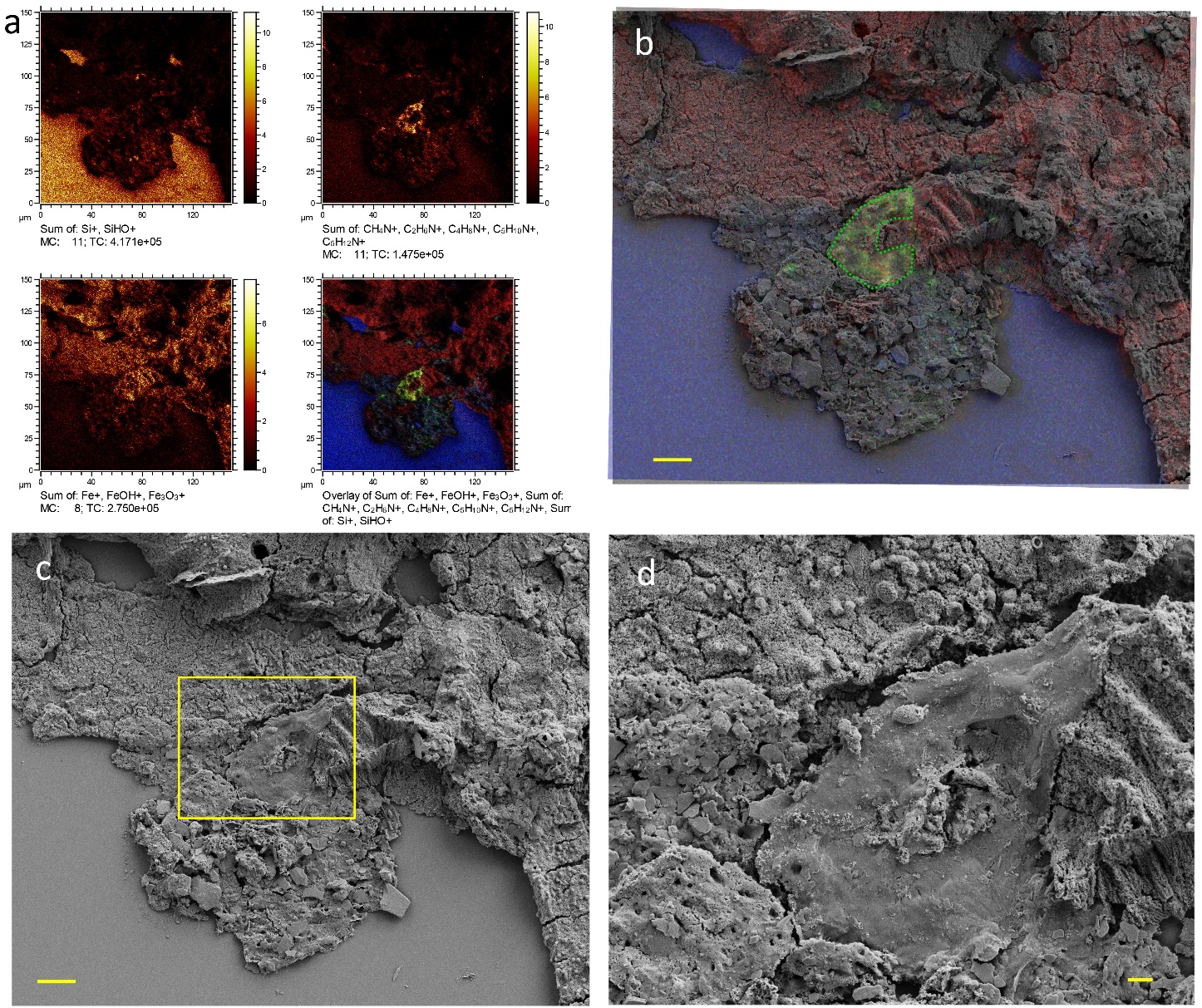


**Fig. S9**. Localization of detected proteins in vessel structure of MOR10857. **a**, ToF-SIMS images of ions representing silicon oxide, proteins, and iron oxide/phosphate, respectively, and an overlay image showing proteins in green, iron phosphate in red, and silicon oxide in blue. **b**, Superimposed overlay ToF-SIMS image (as in **a**) and SEM micrograph of the same area on the vessel surface. The dotted line indicates ROI (S9) from which spectrum was extracted for intensity distribution analysis. **c**, SEM image as in **b**, where the yellow box indicates the area magnified in **d**. **d**, Magnified SEM micrograph highlighting area generating protein fragment ions in ToF-SIMS (flat area, at center). Scale bars, 10 µm (**b-c);** 2 µm (**d)**.


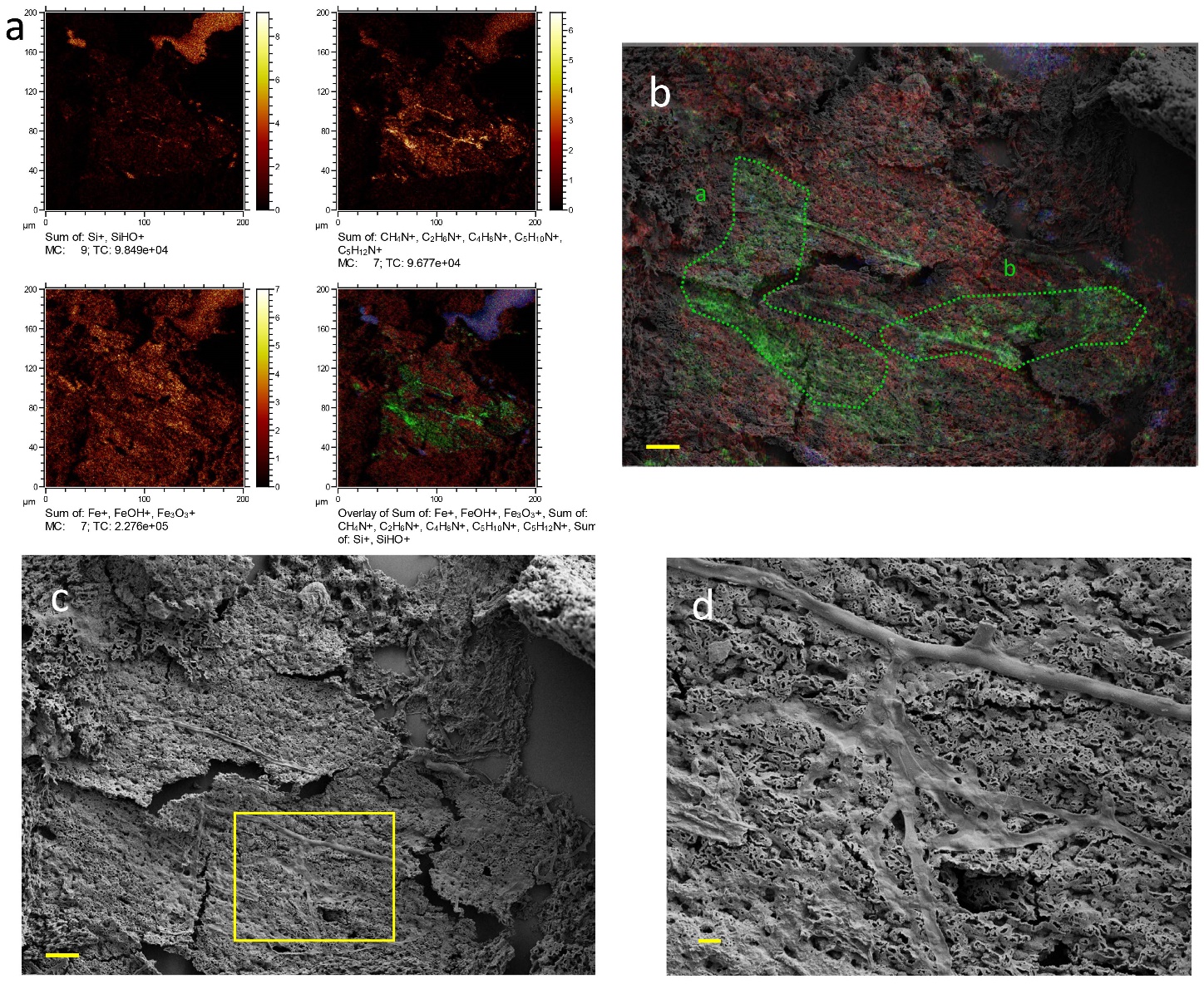


**Fig. S10.** Localization of detected proteins in vessel structure of MOR2598. **a**, ToF-SIMS images of ions representing silicon oxide, proteins, and iron oxide/phosphate, respectively, and an overlay image showing proteins in green, iron phosphate in red, and silicon oxide in blue. **b**, Superimposed overlay ToF-SIMS image (as in **a**) and SEM micrograph of the same area on the vessel surface. The dotted lines indicate ROIs (S10a, S10b) from which spectra were extracted for intensity distribution analysis. **c**, SEM image as in **b**, where the yellow box indicates the area magnified in **d**. **d**, Magnified SEM micrograph highlighting area generating protein fragment ions in ToF-SIMS (elongated structures, resembling fungal hyphae). Scale bars, 10 µm (**b-c);** 2 µm (**d)**.


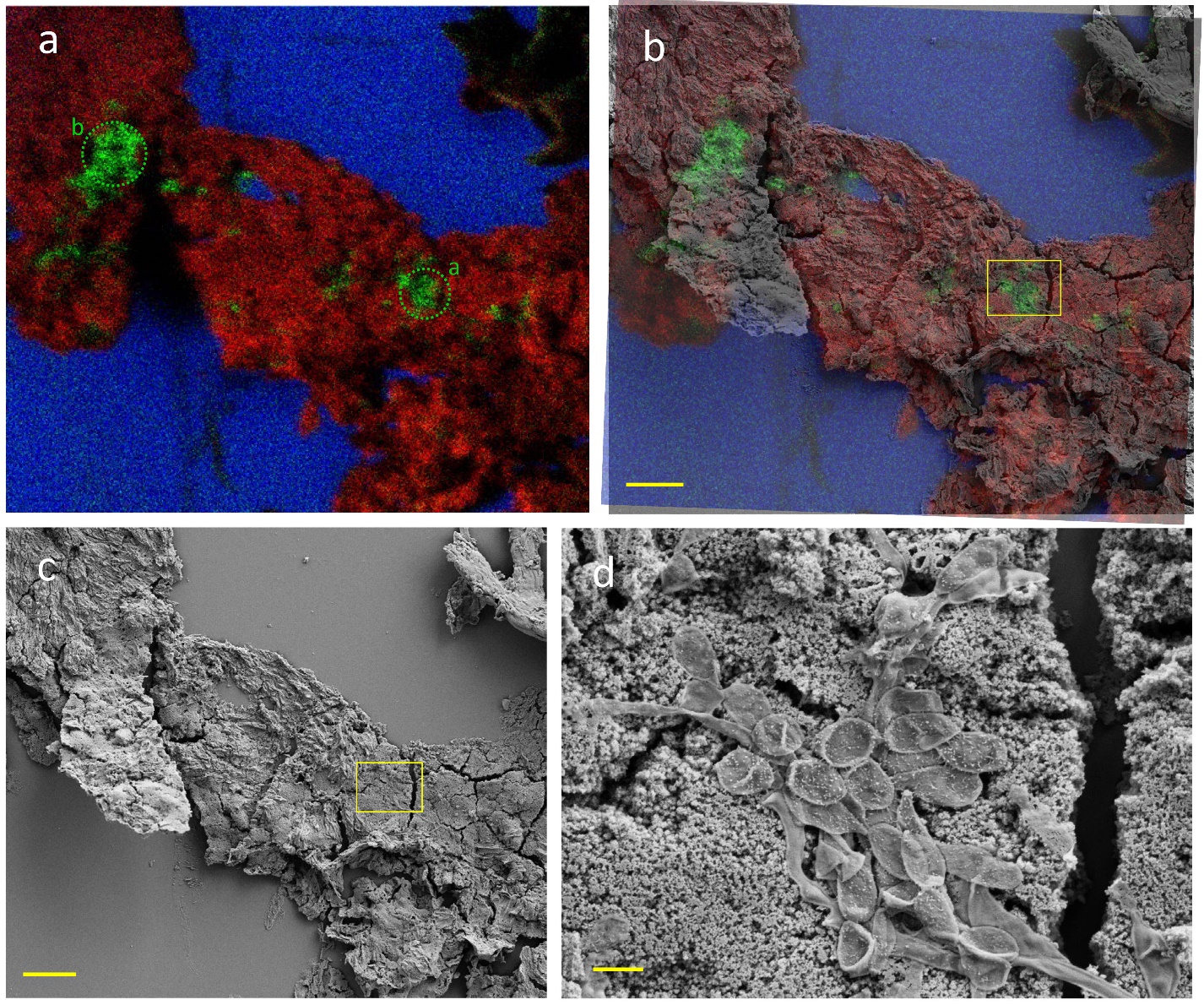


**Fig. S11**. Localization of detected proteins in vessel structure of MOR555. **a**, Overlay ToF-SIMS image of ions representing proteins (green), iron phosphate (red), and silicon oxide (blue). The dotted green lines indicate ROIs (S11a, S11b) from which spectra used for intensity distribution analysis were extracted. **b**, Superimposed ToF-SIMS image (as in **a**) and SEM micrograph of the same area on the vessel surface. **c**, SEM image as in **b**, where the yellow box indicates the area magnified in **d**. **d**, Magnified SEM micrograph of area generating protein fragment ions, displaying structures of likely modern microbial origin. Scale bars, 10 µm (**b-c):** 1 µm (**d)**.


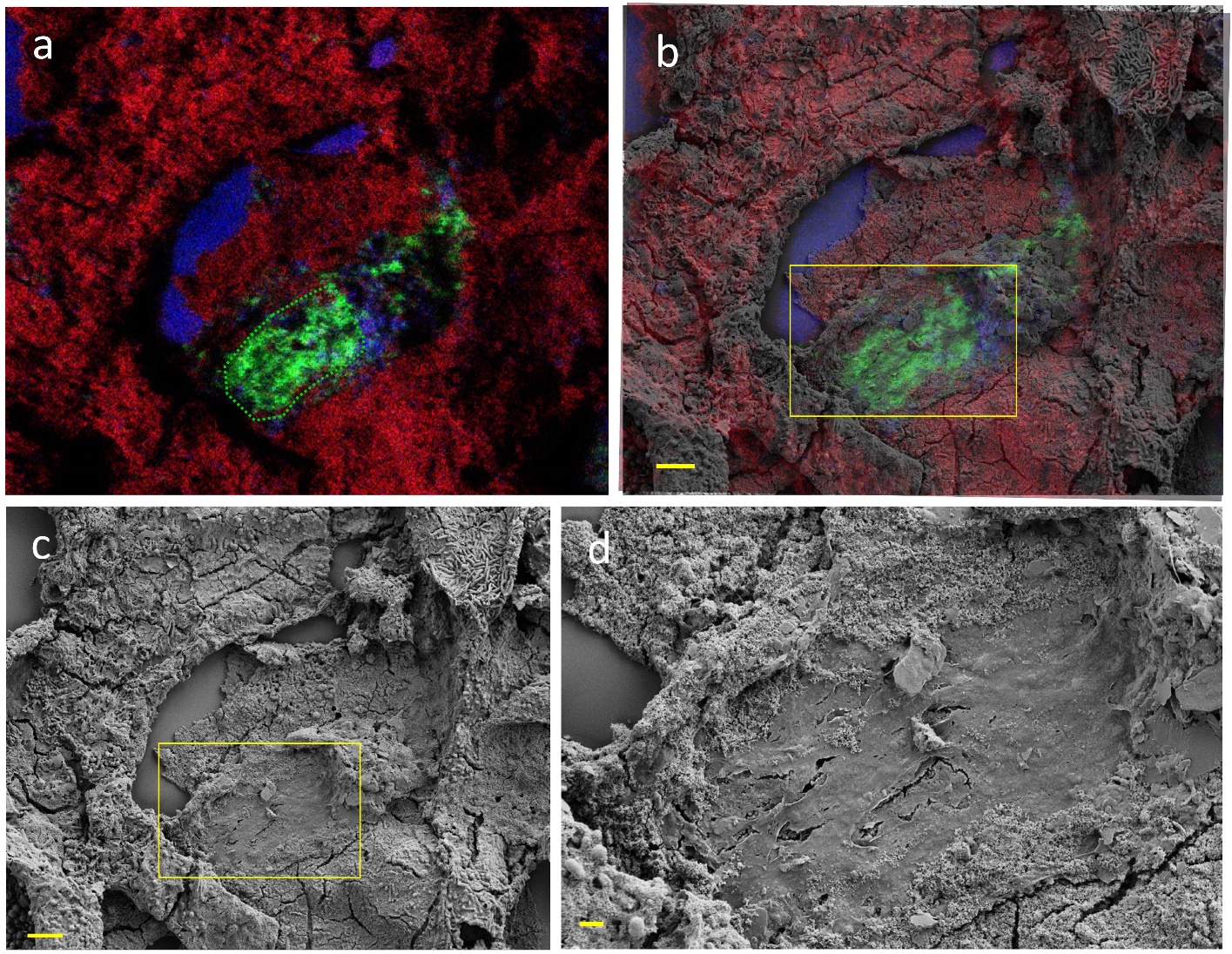


**Fig. S12**. Localization of detected proteins in vessel structure of MOR10857. **a**, Overlay ToF-SIMS image of ions representing proteins (green), iron phosphate (red), and silicon oxide (blue). The dotted green line indicates ROI (S12) from which spectrum used for intensity distribution analysis was extracted. **b**, Superimposed ToF-SIMS image (as in **a**) and SEM micrograph of the same area on the vessel surface. **c**, SEM image as in **b**, where the yellow box indicates the area magnified in **d**. **d**, Magnified SEM micrograph of area generating protein fragment ions, displaying flat area without apparent microbial association. Scale bars, 10 µm (**b-c);** 2 µm (**d)**.

References

1. Schweitzer, M. H. *et al.* A role for iron and oxygen chemistry in preserving soft tissues, cells and molecules from deep time. *Proc. R. Soc. B Biol. Sci.* **281**, 1–10 (2013).

2. Cleland, T. P. *et al.* Mass spectrometry and antibody-based characterization of blood vessels from *Brachylophosaurus canadensis*. *J. Proteome Res.* **14**, 5252–5262 (2015).

3. Eble, J. & Niland, S. The Extracellular Matrix of Blood Vessels. *Curr. Pharm. Des.* **15**, 1385–1400 (2009).

4. Kawecki, M. & Bernard, L. Database of proteinogenic amino acid reference spectra for Bismuth-cluster ToF-SIMS. II. Positive polarity. *Surf. Sci. Spectra* **25**, (2018).
